# Wolf-Hirschhorn syndrome candidate 1 (*Whsc1*) methyltransferase signals *via* a *Pitx2*-*miR-23/24* axis to effect tooth development

**DOI:** 10.1016/j.jbc.2023.105324

**Published:** 2023-10-06

**Authors:** Dan Su, Steve Eliason, Zhao Sun, Fan Shao, Brad A. Amendt

**Affiliations:** 1 Department of Anatomy and Cell Biology, The University of Iowa, Iowa City, Iowa, USA; 2 Craniofacial Anomalies Research Center, Carver College of Medicine, The University of Iowa, Iowa City, Iowa, USA; 3 College of Medicine, Washington University St Louis, St Louis, Missouri, USA; 4 Iowa Institute for Oral Health Research, College of Dentistry, The University of Iowa, Iowa City, Iowa, USA

**Keywords:** miR-23, miR-24, miR-27, PMIS, plasmid-based microRNA inhibitor system, tooth development, dental epithelial stem cells, methyltransferase, Wolf-Hirschhorn syndrome, microRNAs, Pitx2

## Abstract

Wolf-Hirschhorn syndrome (WHS) is a developmental disorder attributed to a partial deletion on the short arm of chromosome 4. WHS patients suffer from oral manifestations including cleft lip and palate, hypodontia, and taurodontism. WHS candidate 1 (*WHSC1*) gene is a H3K36-specific methyltransferase that is deleted in every reported case of WHS. Mutation in this gene also results in tooth anomalies in patients. However, the correlation between genetic abnormalities and the tooth anomalies has remained controversial. In our study, we aimed to clarify the role of *WHSC1* in tooth development. We profiled the *Whsc1* expression pattern during mouse incisor and molar development by immunofluorescence staining and found *Whsc1* expression is reduced as tooth development proceeds. Using real-time quantitative reverse transcription PCR, Western blot, chromatin immunoprecipitation, and luciferase assays, we determined that *Whsc1* and *Pitx2*, the initial transcription factor involved in tooth development, positively and reciprocally regulate each other through their gene promoters. miRNAs are known to regulate gene expression posttranscriptionally during development. We previously reported *miR-23a/b* and *miR-24-1/2* were highly expressed in the mature tooth germ. Interestingly, we demonstrate here that these two miRs directly target *Whsc1* and repress its expression. Additionally, this miR cluster is also negatively regulated by *Pitx2*. We show the expression of these two miRs and *Whsc1* are inversely correlated during mouse mandibular development. Taken together, our results provide new insights into the potential role of *Whsc1* in regulating tooth development and a possible molecular mechanism underlying the dental defects in WHS.

Wolf-Hirschhorn syndrome (WHS) is a genetic disorder that is estimated to affect one in 50,000 births. WHS patients show different levels of symptoms which consist of a characteristic facial appearance, delayed growth and development, intellectual disabilities, and seizures (1, 2, 3, 4, 5). WHS is caused by subtelomeric deletions of the short arm of chromosome 4 (4p) and the variety and severity of the clinical features largely depend on the number and roles of the genes in the deletion (6, 7, 8). In addition to these common features, delayed tooth development and tooth anomalies have also been considered as underestimated traits with a prevalence of 50% in WHS patients (9, 10, 11). WHS candidate 1(*WHSC1*) or nuclear receptor binding SET domain protein 2 (*NSD2*), is a H3K36-specific methyltransferase (12, 13, 14, 15). This gene resides in the “critical region” of WHS and is deleted in every case of WHS (4, 16). Interestingly, it has been reported that patients with nonsense or loss-of-function variant of *WHSC1* also exhibit a subset of WHS features, including intrauterine growth retardation and global developmental delay (17, 18). Interestingly, a patient with *de novo* nonsense mutation in the *WHSC1* gene (c.3412C > T, p.Arg1138Ter, NM_001042424.2) also shows tooth enamel dystrophy (19). *Whsc1*-deficient mice have WHS features including growth retardation, cleft palate, bone defects, congenital heart disease, and malocclusion (15, 20). These reports suggest a potential role of *WHSC1* in regulating tooth development.

Tooth development is a complex process requiring reciprocal interactions between the dental epithelium and mesenchyme, involving bone morphogenetic protein, wingless-related integration site, fibroblast growth factor, and Sonic Hedgehog signaling pathways (21). This cell-cell communication requires rigid spatiotemporal regulation of transcription factors (TFs) to ensure a staged morphogenesis of individual tooth germs (initiation, placode, bud, cap, and bell), as well as odontoblast and ameloblast differentiation, the two unique dental cell types producing dental hard tissues (22). *PITX2* is the earliest TF observed in tooth development, which has long been considered to regulate the transcriptional hierarchy in early stages of tooth development, as well as the stem cell niche (23, 24, 25, 26, 27, 28). It controls dental epithelial stem cell activity by activating several genes, including *Lef-1* and *Sox2* and thus initializes embryonic tooth development and enamel formation (29, 30, 31, 32, 33, 34).

MicroRNAs (miRNAs/miRs) are posttranscriptional regulators that repress gene expression by binding to their specific binding sites in the *3′UTR* region of target mRNAs, and thus regulate biological processes such as cell proliferation, apoptosis, and differentiation (35, 36). They also play important roles in the developing tooth (37, 38, 39, 40, 41). For example, dental epithelial-specific KO of *Dicer1*, which encodes a miR processing enzyme, leads to severe enamel defects and supernumerary incisors (37). *miR-23-27-24* clusters, including *miR-23a-27a-24-2* (mouse chromosome 8) and *miR-23b-27b-24-1* (mouse chromosome 13), encode *miR-23a/b*, *miR-27a/b* and *miR24-1/2*, and their expression are associated with bone development (42, 43, 44), endocrine homeostasis (45), cell death (46), glutamine metabolism (47), and cancer development (48). We reported that miRs from this cluster are highly expressed in the mouse tooth germ at P0 (37). A Solexa sequencing of miR expression profiles in miniature pigs also revealed the high expression of miRs in the *miR-23-27-24* cluster during tooth development (49). We recently described a role for *miR-23a* and *miR-23b* in regulating *Hmgn2*, a chromatin-associated factor that inhibits Pitx2 protein function, during dental epithelial development (50).

In this report, we aimed to determine if *Whsc1* has a role in regulating tooth development. We hypothesized that *Whsc1* participates in the regulatory network involving TFs and miRNAs during mouse lower incisor development. In this work, we describe a new molecular mechanism for *Whsc1*/*Nsd2* in regulating mouse tooth development. Whsc1, as a methyltransferase, positively regulates the *Pitx2* promoter. The *Whsc1* promoter is also regulated by Pitx2. Furthermore, *Whsc1* is directly targeted by *miR-23a/b* and *miR-24-1/2*. The expression of *Whsc1* and *miR-23-27-24* clusters are negatively correlated during development. Interestingly, *miR-23-27-24* clusters are repressed by Pitx2, through interaction of the Pitx2 protein on a distal binding element upstream of the miR clusters.

### Significance

While the developmental and protein function of Whsc1 has been studied, little is known about its specific expression pattern and regulatory role in causing WHS. Our research defines a specific expression and regulatory network between Whsc1, Pitx2, and miRs encoded by the *miR-23-27-24* cluster in tooth development, which also indicates a potential mechanism underlying tooth anomalies in WHS.

## Results

### *Whsc1* is developmentally regulated and associated with mesenchymal and epithelial progenitor cells in the mouse incisor

**Figure 1:**
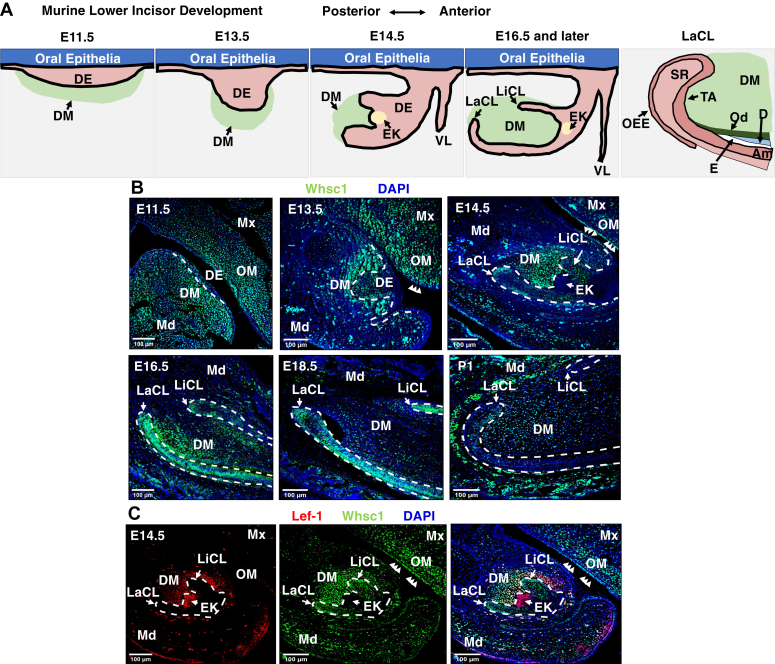
**The expression pattern of *Whsc1* during murine lower incisor development.***A*, Whsc1 immunofluorescence signal in lower incisors (dental epithelium outlined with *dashes*) at E11.5, E13.5, E14.5, E16.5, E18.5, and P1. DAPI staining represents nuclei. *B*, representative images showing immunofluorescence staining of Whsc1 staining from E11.5 to P1. *C*, representative images showing Whsc1 and Lef-1 staining in cap-stage (E14.5) teeth. Regions outlined by *dashes* show cap-stage teeth. *Arrows* indicate Whsc1 signal in oral epithelial cells and Lef-1 expression in the EK. The scale bar represents 100 μm. DAPI, 4′,6-diamidino-2-phenylindole; DE, dental epithelium; DM, dental mesenchyme; EK, enamel knot; LaCL, labial cervical loop; LiCL, lingual cervical loop; Md, mandible; Mx, maxilla; OM, oral mesenchyme; Whsc1, Wolf-Hirschhorn syndrome candidate 1.

We first examined the expression pattern of *Whsc1* during mouse incisor development (Fig. 1). A schematic of the embryonic stages of mouse lower incisor development is shown for reference (Fig. 1*A*). At the placode stage (E11.5), Whsc1 is extensively expressed in the mandible and maxilla. It is more restricted to the dental mesenchyme (DM) and dental epithelium in the mandible, while still expressed ubiquitously in the maxilla at the bud stage (E13.5). At the cap stage (E14.5), when the enamel knot (EK) is formed, Whsc1 expression is limited to the lingual cervical loop, labial cervical loop (LaCL) and the DM in the lower incisor tooth germ. Later at bell stages (E16.5 and E18.5), Whsc1 expression decreases over time. At P1, when the mature tooth germ is formed, Whsc1 expression is further reduced and limited to the transit amplifying zone and some preameloblasts in the LaCL, as well as some dental mesenchymal cells (Fig. 1*B*). By costaining with Lef-1, which marks the EK in the lower incisor tooth germ, we found that Whsc1 was excluded from the EK (Fig. 1*C*). EK is generally recognized as a signaling center that controls the growth of the surrounding epithelium and mesenchyme, while it itself contains a group of nondividing cells (51, 52). Previous studies have reported several cell cycle proteins are expressed in the developing lower incisor tooth germ. The expression pattern of Ki-67 and cyclin A is similar to Whsc1 (53, 54). We also performed immunofluorescence staining of Whsc1 in the developing first molar and found a similar expression pattern compared to the incisor (Fig. S1, *A* and *B*). This specific expression pattern suggests that Whsc1 may be linked to proliferation. To test this hypothesis, we performed cell proliferation assays with LS-8 and LS-8-*NSD2* cells. Both the 3-(4,5-dimethylthiazol-2-yl)-2,5-diphenyl-2H-tetrazolium bromide (MTT) and cell counting assays demonstrate a role of *Whsc1* in activating proliferation in dental epithelial cells (Fig. S1, *C* and *D*).

**Figure 2:**
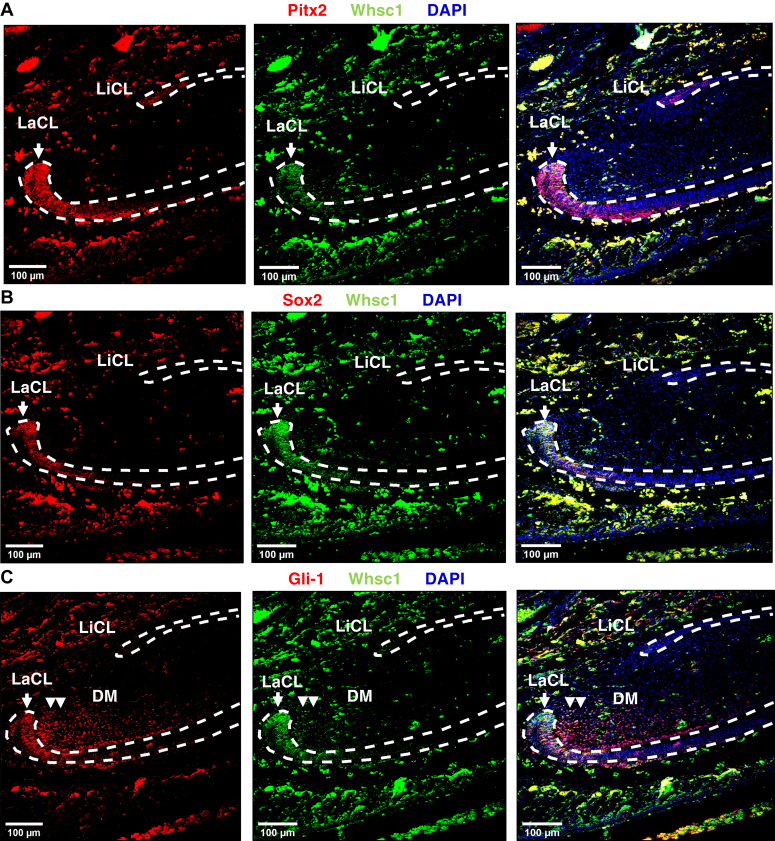
**Whsc1 is expressed in DESCs and DMSCs.***A*, representative images showing immunofluorescence staining of DESC marker Pitx2 (*Red*) and Whsc1 (*Green*) in P0 lower incisor. *B*, representative images showing immunofluorescence staining of DESC marker Sox2 (*Red*) and Whsc1 (*Green*) in P0 lower incisor. *C*, representative images showing immunofluorescence staining of DMSC marker Gli-1 (*Red*) and Whsc1 (*Green*) in P0 lower incisors. The scale bar represents 100 μm. DESC, dental epithelial stem cell; DM, dental mesenchyme; DMSC, dental mesenchymal stem cell; LaCL, labial cervical loop; LiCL, lingual cervical loop; Whsc1, Wolf-Hirschhorn syndrome candidate 1.

We next costained Whsc1 with progenitor cell markers in the P0 (at birth) tooth germ (Fig. 2). We first costained Whsc1 with Pitx2, the first transcription marker observed during tooth development, and found that they colocalize in the LaCL or dental epithelial stem cells (DESCs) (Fig. 2*A*). Another DESC marker, Sox2 shows a similar colocalization with Whsc1 in the DESCs (Fig. 2*B*). Gli-1, a Sonic Hedgehog signaling mediator and a dental mesenchymal stem cell marker (55, 56), colocalizes with Whsc1 in the DESCs and DM (Fig. 2*C*). These data together demonstrate that Whsc1 is associated with the epithelia and mesenchyme progenitor cells.

### Whsc1 and Pitx2 positively and reciprocally regulate their expression

**Figure 3:**
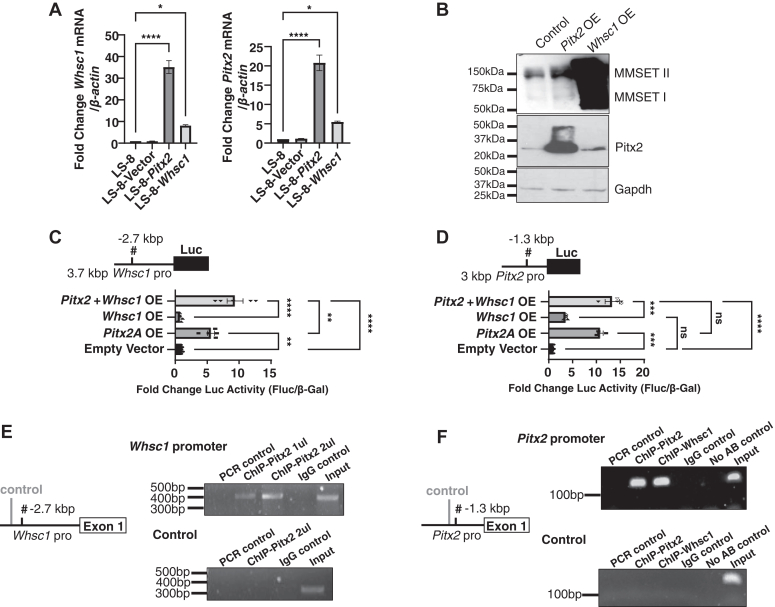
**Whsc1 and Pitx2 positively and reciprocally regulate one another.***A*, RNA isolated from the LS-8, LS-8-Vector, LS-8-*Pitx2*, and LS-8-*Whsc1* overexpressing cells were analyzed by qPCR for *Whsc1* and *Pitx2* transcript levels. Relative levels of each transcript are shown (N = 3). *B*, HEK 293T cells were transfected with control, Pitx2 or Whsc1/Nsd2 expression plasmids and proteins were isolated 48 h after transfection. Isolated proteins were then resolved on a 10% SDS-polyacrylamide gel and probed for Pitx2 and two isoforms of Whsc1 (MMSET I and II). Gapdh was used as internal control. The proteins were visualized using ECL reagents. *C* and *D*, LS-8 cells were cotransfected with the *Whsc1* or *Pitx2* luciferase promoter (5 μg) construct, either Pitx2, Whsc1 or both expression constructs (2.5 μg). The luciferase activities are shown as mean fold activation compared with the normalized luciferase activity in empty vector (pcDNA 3.1) with the *Whsc1* or *Pitx2* promoter reporter (N = 3–5). *E*, schematic of the *Whsc1* promoter chromatin region and ChIP primers. ChIP assay using anti-PITX2^ABC^ Ab for chromatin immunoprecipitations (IP). Pitx2 bound to the Whsc1 distal element in the Whsc1 promoter. IgG alone did not IP the chromatin. PCR and no AB control groups did not produce a band. Control primers to an upstream region of the *Whsc1* promoter did not detect an IP product in any group except the input. *F*, schematic of the *Pitx2* promoter chromatin region and ChIP primers. ChIP assay using either anti-PITX2^ABC^ or anti-WHSC1 Ab for chromatin immunoprecipitations. Pitx2 and Whsc1 bound to the Pitx2 promoter. IgG alone did not IP the chromatin. PCR and no AB control groups did not produce a band. Control primers to an upstream region of the *Pitx2* promoter did not detect an IP product in any group except the input. #, PITX2 binding site. ∗*p* < 0.05; ∗∗*p* < 0.01; ∗∗∗*p* < 0.001; ∗∗∗∗*p* < 0.0001. ChIP, chromatin immunoprecipitation; IgG, immunoglobulin G; IP, immunoprecipitation; MMSET, multiple myeloma SET domain; qPCR, quantitative PCR; Whsc1, Wolf-Hirschhorn syndrome candidate 1.

Because Pitx2 is an early developmental TF and Whsc1 is expressed in early development, we asked if Pitx2 regulates *Whsc1*expression. We first established *Pitx2* and *Whsc1* overexpression stable dental epithelial LS-8 cell lines and performed real-time quantitative reverse transcription PCR (RT-qPCR) (Fig. 3*A*). *Whsc1* mRNA was increased in the LS-8-Pitx2 stable cell line and surprisingly, *Pitx2* mRNA was increased in the Whsc1 stable cell line. We confirmed this regulation by transfection of Pitx2 and Whsc1 in HEK 293 cells. Thus, Pitx2 transfection increased Whsc1 (Multiple myeloma SET domain [MMSET] II and MMSETI) protein and Whsc1 transfection increased Pitx2 protein expression, compared to untransformed cells (Fig. 3*B*).

We next screened the promoter region of the murine *Whsc1* gene and found a Pitx2 binding site (TAATCC) at −2.7 kb, which is also conserved in humans (Fig. 3*C*). The 3.7 kb *Whsc1* promoter was cloned in a luciferase reporter, and the overexpression of Pitx2 expression increased luciferase (luc) activity 6-fold in cells (Fig. 3*C*). While Whsc1 did not increase *Whsc1* promoter luc activity, cotransfection of Pitx2 and Whsc1 increased *Whsc1* promoter luc activity at 9-fold compared to control (Fig. 3*C*). We have previously shown that Pitx2 autoregulates its expression by binding to a *Pitx2* cis-element in the Pitx2 promoter (57). We next asked if Whsc1 could regulate the *Pitx2* promoter. By performing the luciferase assay with the 3 kb *Pitx2* promoter reporter, we show that Pitx2 or Whsc1 can upregulate luc activity by 11-fold and 4-fold, respectively (Fig. 3*D*). Together Whsc1 and Pitx2 show an increase in *Pitx2* promoter activity to 14-fold (Fig. 3*D*). Because Whsc1 is a methyltransferase that methylates H3K36 in the proximal promoters of genes (12, 13, 14, 15, 20), Whsc1 may activate the *Pitx2* promoter by depositing active chromatin marks onto its promoter. A chromatin immunoprecipitation (ChIP) assay using a Pitx2 antibody indicated that Pitx2 binds to its cis-element in the *Whsc1* promoter (Fig. 3*E*). Interestingly, the binding of Pitx2 and Whsc1 at the *Pitx2* promoter were detected by the ChIP assay (Fig. 3*F*), suggesting that Whsc1 is binding to chromatin at the Pitx2 binding element in the *Pitx2* promoter. Moreover, H3K36me2 is also associated with the Pitx2 binding sites in the *Pitx2* and *Whsc1* promoters demonstrated by ChIP assays (Fig. S2). In addition, Whsc1 as well as the H3K36me2 modifications were detected at the Pitx2 binding element in the *Amelogenin* and *Sox2* promoters (Fig. S3).

Taken together, these data indicate that Pitx2 upregulates *Whsc1* expression by binding to and activating its promoter; Whsc1 positively regulates *Pitx2* expression by activating the *Pitx2* promoter, through depositing active chromatin marks around the *Pitx2* proximal promoter. *Whsc1* and *Pitx2* act to positively regulate gene expression through different mechanisms.

### PITX2 represses the expression of the *miR-23-27-24* cluster by binding to an upstream distal regulatory element

**Figure 4:**
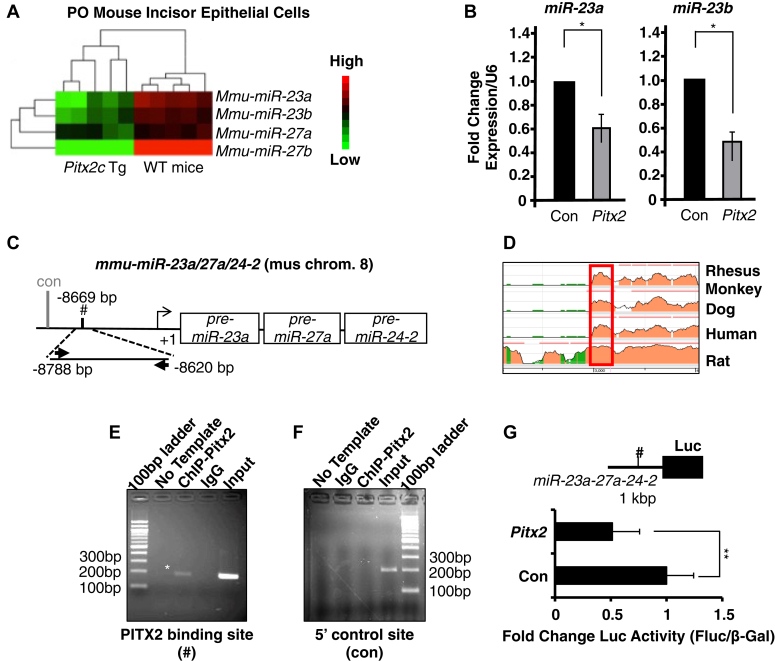
**Pitx2 represses *miR-23a* and *miR-23b* expression.***A*, *miR-23a-27a-24-2* and *23b-27b-24-1* cluster expression are down-regulated in *Pitx2c* transgenic mice. microRNA array (heat map) shows the expression levels of *miR-23a/b* families in *Pitx2c*-transgenic and WT P0 mice. *B*, Pitx2 represses endogenous *miR-23a* and *miR-23b* in epithelial cells. LS-8 cells were cotransfected with 2.5 μg of either the pcDNA-*Pitx2*, or the empty vector pcDNA3.1 (control) plasmids. The *miR-23a* or *miR-23b* expression levels in cells transfected with Pitx2 were normalized to cells transfected with empty vector (N = 3). *C*, schematic representation and location of the *Pitx2* binding site in the *pre-miR-23a-27a-24-2* promoter. Pound sign indicates the region containing a conserved *Pitx2* binding element (TCATCC). The *gray line* (con) indicates a 5′ region which lacks Pitx2 consensus binding motif and was used as negative control. *D*, the Pitx2 binding element of the mouse pre-*miR-23a-27a-24-2* promoter was mapped to a highly conserved region among mouse, monkey, dog, human, and rat. The *red box* indicates the PCR amplified region on pre-*miR-23a-27a-24-2* promoter in (*C*). *E*, ChIP-PCR assay of endogenous Pitx2binding to the chromatin region approximately 8600 bp upstream of pre-*miR-23a-27a-24-2* transcript in LS-8 cells. *F*, control ChIP-PCR assay using the Pitx2 antibody and primers to a 9.9 kb upstream region of the pre-*miR-23a-27a-24-2* transcript. This chromatin region does not contain a Pitx2 binding site. *G*, inhibition of the *miR-23a-27a-24-2* cluster by Pitx2. 1 kb *miR-23a-27a-24-2* enhancer fragment which contains this binding element was cloned into the pTK-Luc reporter vector. LS-8 cells were transfected with 5 μg *miR-23a-27a-24-2* promoter luciferase reporter constructs. The cells were cotransfected with 2.5 μg of either the pcDNA-*Pitx2*, or the empty plasmid as a control (pcDNA3.1). Pound sign indicates the region containing a conserved *Pitx2* binding element (TCATCC) (N = 3). ∗*p* < 0.05; ∗∗*p* < 0.01. ChIP, chromatin immunoprecipitation.

miRNAs are known to regulate tooth development. We asked if a discrete group of miRs participates in this *Whsc1*-*Pitx2* regulation of tooth development. Our lab has previously performed miRNA arrays in P0 mouse tooth germs and revealed the miR expression profile in the late-stage development of mouse incisors and molars (37). We also performed a miR array in the P0 incisor epithelium of WT and *Pitx2c*^*Tg*^ mice to detect miRs regulated by Pitx2. Interestingly, we found that miRs from the *miR-23-27-24* cluster (*miR-23a/b*, *miR-24-1/2* and *miR-27a/b*) are highly expressed in both incisors and molars. Moreover, their expressions are downregulated in the P0 incisor epithelium of *Pitx2c*^*Tg*^ mice (Fig. 4*A*). We then confirmed that *miR-23a* and *miR-23b* expressions were decreased by Pitx2 in transfected LS-8 cells (Fig. 4*B*). We found a highly conserved Pitx2 binding site approximately 8.7 kb upstream of the transcription start site (TSS) of *pre-miR-23a-27a-24-2* (Fig. 4, *C* and *D*). We performed a ChIP assay with the anti-PITX2 antibody and confirmed the binding of Pitx2 at this specific genomic region (Fig. 4*E*, see asterisk, and *F* control). We then cloned a 1 kb region containing the Pitx2 binding element into a luciferase reporter plasmid and performed luciferase assay. The result showed that overexpression of *Pitx2* can repress the luciferase activity from the miR promoter by 50% (Fig. 4*G*). There is also a highly conserved Pitx2 binding element at 73 kb upstream of *pre-miR-23b-27b-24-1* (Fig. S4, *A* and *B*). The binding of Pitx2 at this element was confirmed by ChIP assay (Fig. S4, *C* and *D*). These data indicate that Pitx2 can repress the expression of tooth-development-related *miR-23-27-24* clusters by interacting with upstream distal regulatory elements.

### *miR-23a/b* and *miR-24-1/2* regulate *Whsc1* expression

**Figure 5:**
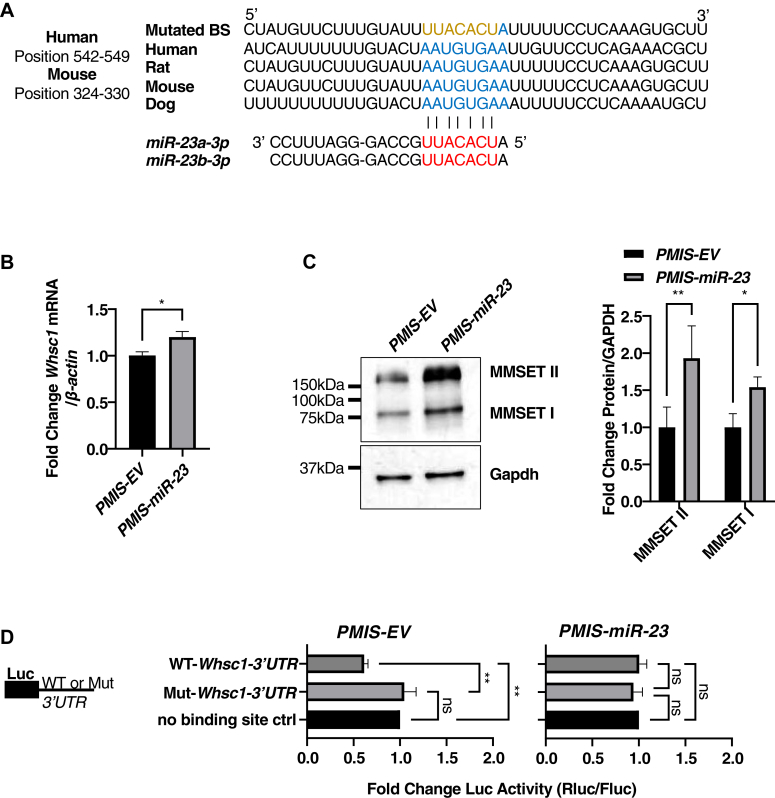
***miR-23a/b-3p* directly targets and inhibits *Whsc1*.***A*, schematics showing the potential *miR-23a/b-3p* binding site in the *Whsc1 3′UTR*, which is highly conserved among different species. *Red* ribonucleotides represent the seed sequence of *miR-23a/b-3p*; *blue* ribonucleotides represent the potential binding sites; and *gold* ribonucleotides represent the mutated binding site used in the luciferase reporter. *B*, RT-qPCR shows *Whsc1* is upregulated in the LS-8-*PMIS-miR-23* stable cell line. The expression of *Whsc1* mRNA is normalized to *β-actin*. Data are represented as fold change against LS-8-*PMIS-EV*. *C*, the two main protein isoforms of WHSC1: MMSET I and MMSET II, are upregulated in the LS-8-*PMIS-miR-23* stable cell line. Western blots were quantified using Fiji ImageJ. The band intensity of MMSET I and II were normalized to Gapdh. Data represent the fold change against LS-8-*PMIS-EV*. *D*, *miR-23a/b-3p* directly targets the WT but not the mutated mouse *Whsc1 3′UTR*. The psiCheck2 reporter with either no binding site control, WT or MT *Whsc1 3′UTR* (5 μg) were transfected to both LS-8-*PMIS-EV* and LS-8-*PMIS-miR-23*. Cells were incubated for 48 h and then assayed for Firefly and Renilla luciferase activity (N = 5–7). Data were shown as fold change against no binding site control group. ∗*p* < 0.05; ∗∗*p* < 0.01. MMSET, multiple myeloma SET domain; Mut, mutant; PMIS, plasmid-based miRNA inhibition system; RT-qPCR, real-time quantitative reverse transcription PCR; Whsc1, Wolf-Hirschhorn syndrome candidate 1.

We analyzed the *3′UTR* of the *Whsc1* transcript and observed a conserved binding site for *miR-23a/b-3p* (Fig. 5*A*). To determine if *miR-23a/b-3p* negatively regulates *Whsc1* expression, we utilized our previously developed plasmid-based miRNA inhibition system (PMIS) and established stable cell lines that can specifically inhibit *miR-23a/b* expression without affecting *miR-27a/b* or *miR-24-1/*2 levels (Fig. S5*A*). In the *PMIS-miR-23* LS-8 cell line, both mRNA and protein levels of *Whsc1* gene (MMSETI and MMSET II) were elevated (Fig. 5, *B* and *C*). By cloning the *3′UTR* of *Whsc1* containing either the WT or mutated *miR-23-a/b* binding sites into our luciferase reporter, we found that endogenous *miR-23-a/b* can only bind to the WT *3′UTR* to reduce luciferase activity (50%) in the *PMIS-EV* LS-8 cell line (Fig. 5*D*). However, this inhibition can be recovered when endogenous *miR-23-a/b* was inhibited by *PMIS-miR-23* in the LS-8 cell line (Fig. 5*D*).

**Figure 6:**
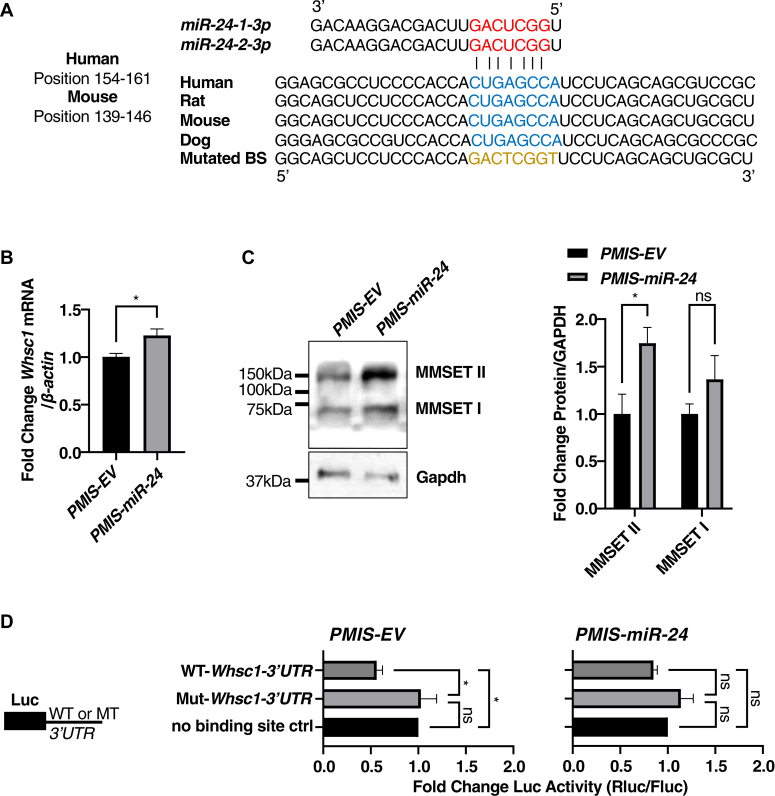
***miR-24-1/2-3p* directly targets and inhibits *Whsc1*.***A*, schematics showing the potential *miR-24-1/2-3p* binding sites in the *Whsc1 3′UTR*, which are highly conserved among different species. *Red* ribonucleotides represent the seed sequence of *miR-24-1/2-3p*; *blue* ribonucleotides represent the potential binding sites; and *gold* ribonucleotides represent the mutated binding site used in the luciferase reporter. *B*, RT-qPCR shows *Whsc1* is upregulated in the LS-8-*PMIS-miR-24* stable cell line. The expression of *Whsc1* mRNA is normalized to *β-actin*. Data are represented as fold change against LS-8-*PMIS-EV*. *C*, the two main protein isoforms of WHSC1; MMSET I, and MMSET II are upregulated in the LS-8-*PMIS-miR-24* stable cell line. Western blots were quantified using Fiji ImageJ. The band intensity of MMSET I and II were normalized to Gapdh. Data represent the fold change against LS-8-*PMIS-EV*. *D*, *miR-24-1/2-3p* directly targets the WT but not the mutated mouse *Whsc1 3′UTR*. The psiCheck2 reporter with either no binding site control, WT or Mut *Whsc1 3′UTR* (5 μg) were transfected to both LS-8-*PMIS-EV* and LS-8-*PMIS-miR-24*. Cells were incubated for 48 h and then assayed for Firefly and Renilla luciferase activity (N = 5). Data were shown as fold change against no binding site control group. ∗*p* < 0.05. BS, binding site; MMSET, multiple myeloma SET domain; Mut, mutant; PMIS, plasmid-based miRNA inhibition system; RT-qPCR, real-time quantitative reverse transcription PCR; Whsc1, Wolf-Hirschhorn syndrome candidate 1.

*miR-24-1/2-3p*, a miRNA from the same cluster as *miR-23-a/b* also targets *Whsc1* (Fig. 6*A*). *PMIS-miR-24* LS-8 cell line specifically inhibits *miR-24-1/2* without affecting *miR-23-a/b* or *miR-27-a/b* (Fig. S5*B*). Both mRNA and protein levels of *Whsc1* were upregulated upon *miR-24-1/2* inhibition of *miR-24* (Fig. 6, *B* and *C*). We also confirmed that endogenous *miR-24-1/2* can decrease the *Whsc1* 3′UTR reporter luciferase activity (∼50%) by binding to the WT *3′UTR* in *PMIS-EV* LS-8 cell line, while inhibition of *miR-24-1/2* recovers the luciferase activity in the *PMIS-miR-24* LS-8 cell line (Fig. 6*D*). Whsc1 is a histone methyltransferase that deposits methyl groups to histones to maintain open chromatin (12, 13, 14, 15). To assess if the function of the Whsc1 protein was affected by *miR-23-a/b* or *miR-24-1/2*, we performed Western blotting and found the expression of total H3K36me1 and H3K36me2 were increased by *miR-23-a/b* inhibition or *miR-24-1/2* inhibition (Fig. S6).

**Figure 7:**
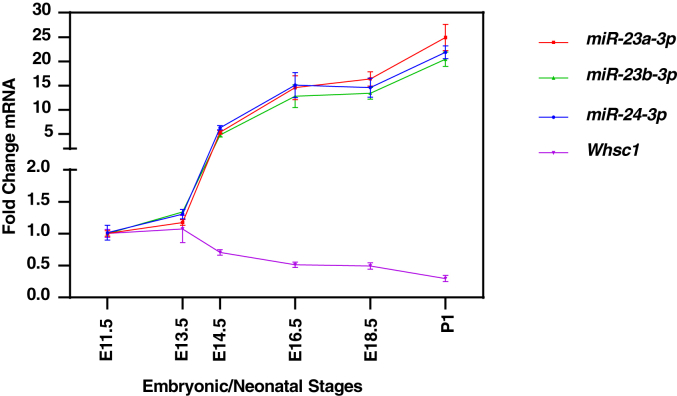
***Whsc1* negatively correlates with *miR-23-3p* and *miR-24-3p* expression during tooth development.** Mouse embryos were harvested at different stages during tooth development. The mandibular RNAs were collected and used for qRT-PCR. The *miR-23a/b* or *miR-24-1/2* expression levels were normalized to *U6* and *Whsc1* expression was normalized to *β-actin*. Data at later time points were represented as fold change against E11.5 (N = 3). qRT-PCR, quantitative real-time PCR; Whsc1, Wolf-Hirschhorn syndrome candidate 1.

Endogenous *Whsc1*, *miR-23-a/b*, and *miR-24-1/2* expression were analyzed during murine mandible/tooth development. Mandibular RNA, including incisor and molar RNA, was collected during key tooth development stages (E11.5, E13.5, E14.5, E16.5, E18.5, and P1) and gene/miR expression was assessed by RT-qPCR. The result showed that *Whsc1* decreased while *miR-23-a/b* and *miR-24-1/2* expression increased, which correlates with the expression pattern of *Whsc1* in Figure 1 and Fig. S1 (Fig. 7). These data suggest that *miR-23-a/b* and *miR-24-1/2* directly target and negatively regulate *Whsc1* during embryonic development. The expression of *Whsc1* is negatively correlated with *miR-23-a/b* and *miR-24-1/2* during mandible/tooth development.

## Discussion

**Figure 8:**
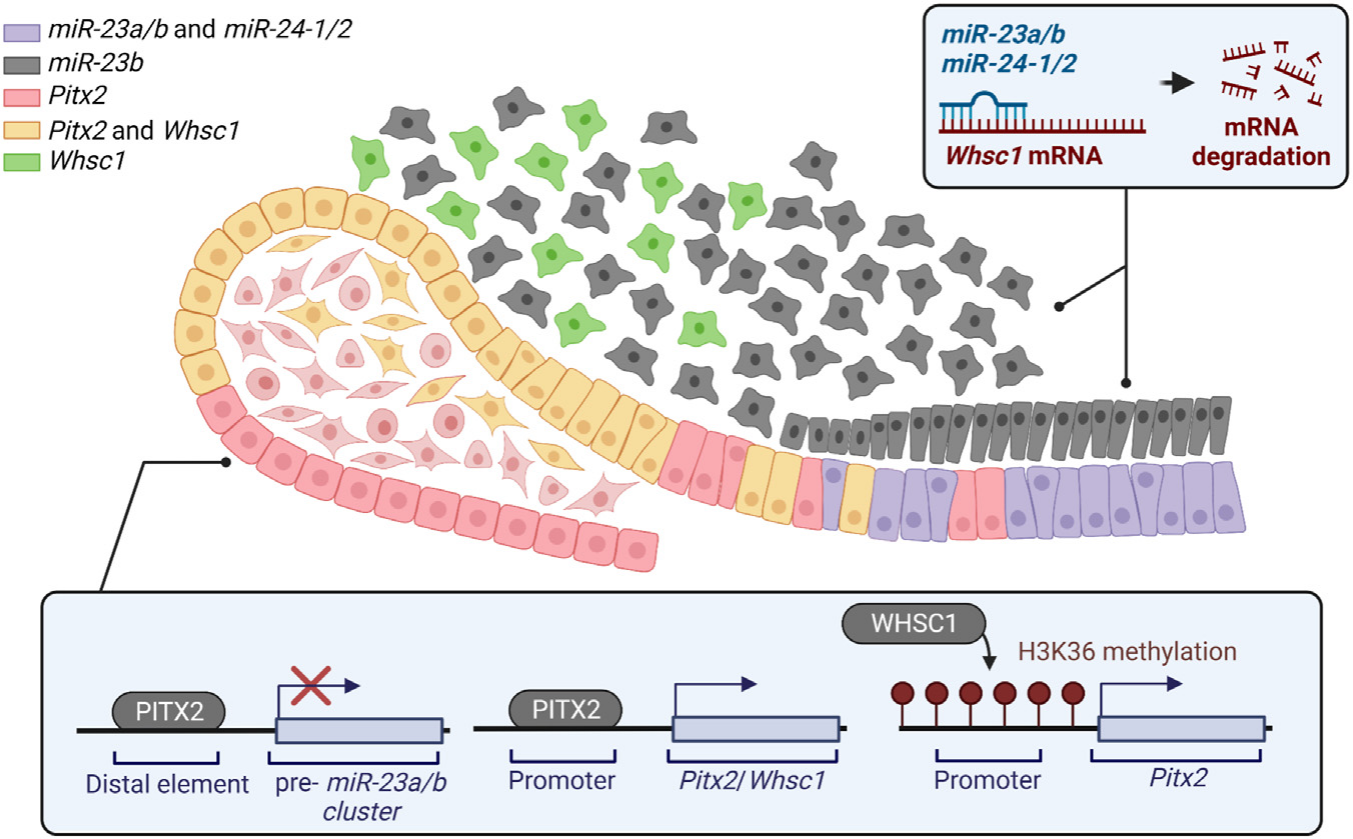
**Model for the role of *Whsc1*, *Pitx2*, *miR-23a/b*, and *miR-24* in the regulation of tooth development.** In the postnatal mouse lower incisor, *Whsc1* is coexpressed with *Pitx2*, in the LaCL, the transit amplifying zone of inner enamel epithelium, and stellate reticulum. In the *Whsc1+/Pitx2+* epithelial cells, *Pitx2* activates *Whsc1* expression by binding to its promoter, but inhibits *miR-23/27/24* cluster expression by binding to its enhancer; *Whsc1* activates the *Pitx2* promoter by depositing the active chromatin mark H3K36me1. In the dental mesenchyme, *Whsc1* is also expressed in the *Gli-1*+ dental mesenchymal stem cells. *miR-23a/b* and *miR-24-1/2* are expressed in the more differentiated dental mesenchyme and epithelial cells, with *miR-23b* expressed in the mesenchyme and all of them expressed in the epithelium (37). In those cells, *miR-23a/b* and *miR-24-1/2* bind to the *3′-UTR* regions of *Whsc1* and inhibit its expression and function. LaCL, labial cervical loop; Whsc1, Wolf-Hirschhorn syndrome candidate 1.

Epigenetic regulation of gene expression during development is fundamental to tissue and organ specific development. These processes have been well-documented and provide a level of gene expression control that supports cell proliferation and differentiation (58, 59, 60, 61). Epigenetic dysfunction is associated with cancer and developmental anomalies such as WHSC1 (MMSET/NSD2) (62). miRNAs regulate gene expression posttranscriptionally and combined with epigenetic factors they offer a mechanism to spatially and temporarily modulate gene expression during different cell processes such as transcription and translation (63). In this report, we have identified a new gene expression regulatory mechanism where Whsc1, a methyltransferase, modulates H3K36 at proximal promoters. However, *Whsc1* expression is also regulated by *miR-23a/b* and *miR-24-1/2*. We demonstrate new mechanisms for *Whsc1* in regulating murine tooth development (Fig. 8).

### *Whsc1* regulates tooth development and underlying mechanisms of tooth anomalies in WHS

Tooth anomalies are an underrepresented feature of WHS, with a prevalence of 50% among WHS patients. A candidate gene Msh Homeobox 1 (*MSX1*), which is located 4.9 Mb from the telomere of 4p short arm, is highly associated with tooth agenesis in WHS, including oligodontia (10, 64). However, patients with small deletions (less than 3 Mb) were also found exhibiting delayed tooth eruption and oligodontia, indicating alternative mechanisms underlying the tooth agenesis in WHS (65, 66). *WHSC1* is deleted from every case of WHS. We have demonstrated that *Whsc1* is expressed in the mouse developing tooth germ, and its expression becomes more restricted during embryonic development. At birth (P0) Whsc1 expression is localized within the LaCL or dental epithelial stem cells and the adjacent DM stem cell region and transient amplifying cells of the lower incisor. These expression profiles suggest that Whsc1 regulates a progenitor cell niche at later stages of tooth development and associated with undifferentiated cell types.

*PITX2* is associated with Axenfeld-Rieger syndrome (ARS), a rare autosomal dominant disorder characterized by a series of craniofacial malfunctions primarily affecting the eye and tooth (23, 24). This novel mechanism of *Whsc1* and *Pitx2* reported in this article has not only broadened our understanding of WHS but has also extended its implications to ARS. Moreover, it also indicates that a potential shared pathway or regulatory network that may contribute to the manifestation of WHS, giving further insights to the etiology of both WHS and ARS.

### *Whsc1* is associated with proliferating cells in the tooth germ

We show that at E14.5 *Whsc1* is expressed in both LaCL and lingual cervical loop epithelial cells but excluded from the EK, which contains cells that are nonproliferative and apoptotic (67, 68).This expression pattern is identical to that of several cell cycle markers, including Ki67 and cyclin A (53, 54). Our cell proliferation assay indicates that Whsc1 activates dental epithelial cell proliferation. *Whsc1* has also been reported to positively regulate cell proliferation (69, 70, 71). These findings demonstrate a potential role of *Whsc1* in controlling cell proliferation during tooth development.

### *Whsc1* expression is controlled by miRNAs

*Whsc1* expression begins at the early stage of mouse embryogenesis and decreases during development. In the mature incisor tooth germ at P0, *Whsc1* expression is restricted to the *Sox2*^*+*^ cells in the LaCL and *Gli-1*^*+*^ cells in the surrounding mesenchyme. The spatial-temporal regulation of gene expression during tooth development is also known to be regulated by miRs. We have shown that *Whsc1* is directly targeted by *miR-23a/b* and *miR-24-1/2*. The expression of *miR-23a/b* and *miR-24-1/2* increases as tooth development proceeds, which negatively correlated with the expression of *Whsc1*. According to the previous microarray, *miR-23b* is highly expressed in both the mouse P0 incisor epithelium and mesenchyme, while *miR-23a* and *miR-24* are more enriched in the epithelium (37). We recently reported that *miR-23a/b* regulates ameloblast differentiation by targeting *Hmgn2* to inhibit its function (50). Hmgn2 is a chromatin-associated high mobility group protein that binds chromatin and modulates transcriptional activity (72). Hmgn2 inhibits several TFs from binding DNA and this prevents the activation of gene expression. These data demonstrate a new mechanism where *miR-23a/b* and *miR-24* are expressed in the more differentiated cell types in the tooth germ, which controls the ameloblast and odontoblast differentiation through epigenetic factors.

### The role of *Whsc1* in regulating gene expression

*Whsc1*, also known as *Nsd2*, encodes a H3K36-specific methyltransferase. This chromatin marker is highly associated with open chromatin, which is mostly enriched in the proximity to the TSS of a gene and recruits RNA Pol II machinery to activate transcription (12, 13). It has been reported that Whsc1 collaborates with TFs to fine-tune the expression of the downstream targets, in regulating heart and bone development (15, 20). We show new roles for *Whsc1* in regulating the *Pitx2* promoter and its downstream targets *Sox2* and *Amelogenin*. Interestingly, we did not observe a direct interaction between Whsc1 and Pitx2 (data not shown), which indicates an indirect interaction between these two proteins or alternatively, an indirect regulatory relationship between these two genes. There are genes that have been reported to interact with the Pitx2 protein and activate its DNA binding and transcriptional activity (31, 32, 73, 74, 75). Whsc1 could activate Pitx2 through chromatin modulation at the *Pitx2* proximal promoter, or *Whsc1* can activate the expression of gene(s) that can increase *Pitx2* transactivation activity.

In summary, in the postnatal mouse lower incisor, *Whsc1* is coexpressed with *Pitx2*, a dental epithelial stem cell marker, in the transit amplifying zone of inner enamel epithelium, stellate reticulum, as well as some pre-ameloblasts in the LaCL. In the *Whsc1+/Pitx2+* epithelial cells, Pitx2 activates *Whsc1* expression by binding to its promoter, but inhibits *miR-23/27/24* cluster expression by binding to its distal regulatory element; Whsc1 activates the *Pitx2* promoter by depositing the active chromatin mark H3K36me1/2. In the dental mesenchyme, *Whsc1* is also expressed in the *Gli-1*+ dental mesenchymal stem cells. *miR-23a/b* and *miR-24-1/2* are expressed in the more differentiated DM and epithelial cells, with *miR-23b* expressed in the mesenchyme and all of the *miR-23/27/24* cluster is expressed in the epithelium (37). In those cells *miR-23a/b* and *miR-24-1/2* bind to the *3′-UTR* regions of *Whsc1* and inhibit its expression and function. Thus, we show how Whsc1 is required for early progenitor cell propagation and the later expression of *miR-23a/b* and *miR-24-1/2* is required for cell differentiation by reducing Whsc1 expression.

## Experimental procedures

### Mouse strain breeding and embryonic staging

All animals were housed, and all procedures performed in accordance with the guidelines approved by the University of Iowa Office of Animal Care. All experimental procedures were approved in accordance with the University of Iowa IACUC guidelines. For embryonic staging experiments, the observed vaginal plug date of the female was designated as E0.5. Embryos were collected at the required time point. Embryos were subjected to tissue fixation or mandibular RNA extraction right after harvesting. The *Krt14-PITX2C* transgenic line has been described previously (76).

### Immunofluorescence staining and histology

Murine embryos and postnatal pups were fixed in 4% paraformaldehyde (ChemCruz, Santa Cruz Biotechnology) and taken through a standard dehydration before being embedded in the paraffin. Samples were sectioned at 7 μm with a Thermo (HM325) microtome as previously reported (33). Sections were subjected to standard H&E staining protocols. Slides with paraffin sections were subjected to a series of dewaxing and rehydration steps and then followed by citric acid antigen retrieval in a 100 °C water bath for 12 min. The slides were then blocked with 20% donkey serum and incubated with primary antibodies overnight at 4 °C. Slides were washed with 1xPBS, incubated with Alexa Fluor 488 and 594 secondary antibodies (Thermo Fisher Scientific), and stained with 4′,6-diamidino-2-phenylindole. Confocal pictures were taken with a ZEISS 700 confocal microscope and Zen imaging software. The primary antibodies are listed in Table 1.

**Table 1:** List of antibodies for immunofluorescence

Antibodies	Manufacturer	Working Dilution
Anti-WHSC1/NSD2 mouse antibody (29D1) ab75359	Abcam	1:50
Anti-PITX2 sheep antibody AF7388	R&D Systems	1:50
Anti-LEF-1 rabbit antibody 2230S	Cell Signaling	1:50
Anti-GLI-1 rabbit antibody ab49314	Abcam	1:50
Anti-SOX2 goat antibody AF2018	R&D Systems	1:50

### Expression and luciferase reporter constructs

A 3.7 kb DNA fragment including the PITX2 binding site upstream of mouse *Whsc1* TSS was cloned into the pTK-Luc vector using primers 5′-TAAGCAGGGATCCGATCTGGAGTCCTGTTTAAT-3′, 5′-TGCTTTAGCGATATCTCTAGCCTCTAGGGG-3′, 5′-TAAGCAGGATATCAGATCTTCCCAAATCAGATCT-3′ and 5′-TGCTTTAGCAAGCTTCCAGCCTAGATCCTTTGG-3′; this vector construct uses the minimal TK promoter (73). A 1 kb DNA fragment containing the PITX2 binding site upstream of either the *miR-23a-27a-24-2* cluster, or the *miR-23b-27b-24-1* cluster was cloned into the pTK-Luc vector. A luciferase reporter plasmid containing the 3 kb DNA fragment upstream of mouse *Pitx2* was used for luciferase assays as previously described (57).

A 60bp-long DNA fragment of the *Whsc1* 3′UTR containing either WT (5′-AATGTGA-3′) or mutant (5′-TCACATT-3′) *miR-23-a/b-3p* binding site was ligated downstream of a Renilla luciferase gene in psiCHECK-2 Vector (Promega). A 60bp-long DNA fragment of *Whsc1* 3′UTR containing either WT (5′-CTGAGCC-3′) or mutant (5′-GGCTCAG-3′) *miR-24-1/2-3p* binding site was ligated downstream of a Renilla luciferase gene in psiCHECK-2 Vector (Promega).

*Pitx2* expression construct has been previously reported (31, 32, 50, 76, 77, 78, 79). *Whsc1/Nsd2* expression construct (LVXN-Neo-NSD2) was a gift from Darrin Stuart (Addgene plasmid # 86010; http://n2t.net/addgene:86010; RRID:Addgene_86010) (80). All the cloned constructs were confirmed by DNA sequencing. All plasmids used for transfection were purified by double-banding in CsCL.

Our PMIS inhibitor constructs *PMIS-miR-23* and *PMIS-miR-24* were cloned as previously described (81).

### Cell culture, transfections, and reporter assays

LS-8 (82), and HEK 293 cells were cultured in Dulbecco's modified Eagle's medium (DMEM) supplemented with 10% fetal bovine serum (FBS) and 1% penicillin/streptomycin. *PITX2*, *WHSC1/NSD2*, *PMIS-EV*, *PMIS-miR-23*, and *PMIS-miR-24* expression plasmids were transfected into LS-8 or HEK 293 cells by either PEI or Lipofectamine 2000 (Invitrogen) reagents, followed by RT-qPCR and Western blot assays. For luciferase reporter assays, cells were seeded 24 h before transfection in 60 mm petri dishes and transfected with 2.5 μg of expression plasmid, 5 μg of reporter plasmid and 0.2 μg of SV-40 β-galactosidase plasmid. Cell transfections were performed by either PEI or Lipofectamine 2000 (Invitrogen) reagents with a DNA:PEI/Lipofectamine 2000 ratio of 1:3 or 1:2. Transfected cells were incubated in 60 mm culture dishes for 48 h and fed with 10% FBS and DMEM. Following lysis with either Reporter Lysis 5X Buffer (Promega) or radioimmunoprecipitation assay buffer, assays for reporter activity (luciferase assay, Promega) as well as for protein concentration (Bradford assay, Bio-Rad) were carried out. β-galactosidase was measured using the Galacto-Light Plus reagents (Tropix Inc) as an internal normalizer. All luciferase activities were normalized to β-galactosidase activity and are shown as mean-fold differences relative to empty luciferase plasmids and are shown as mean ± SEM.

### Lentiviral production and stable cell line establishment

HEK 293T cells were seeded in a 100 mm cell culture dish followed by PEI transfection with pMD2.G, psPAX2 and *PMIS-EV*, *PMIS-miR-23a*, *PMIS-miR-24*, *PITX2*, or *NSD2* expression plasmids. The medium was changed 24 h post transfection and lentivirus-containing medium was collected at 48 h, 72 h, and 96 h post transfection and filtered through a 0.45 μm polyvinylidene fluoride filter. LS-8 cells were seeded in 100 mm cell culture dishes. After 24 h, the medium was aspirated and replaced by lentiviral-containing medium with 8 μg/ml polybrene. Medium was changed to normal culture medium after 24 h. Transduced cells were either selected through puromycin (1 μg/ml) selection, G418 (5 mg/ml) selection or cell sorting. For cell sorting, the cells were trypsinized, washed and resuspended with PBS, and filtered through a 70 μm nylon mesh strainer. The GFP^+^ cell populations were sorted through either Becton Dickinson FACS Aria II or FACS Fusion cell sorters. The sorted cells were then cultured in normal cell culture medium for stable cell line expansion.

### MTT assay

LS8 or LS8-*NSD2* cells were seeded in triplicates for each assay time points in 96-well plates at 20,000 cells/well and cultured in DMEM (10% FBS and 1% penicillin/streptomycin). MTT assay was performed at 6 h, 24 h, and 48 h post seeding. For each MTT assay, media were discarded from cell cultures and 50 μl of serum-free media and 50 μl of MTT solution (5 mg/ml in PBS) were placed into each well. Plates were incubated for 3 h at 37 °C. After incubation, 150 μl MTT reagent (4 mM HCl, 0.1% NP40 in isopropanol) was added to each well. The plate was then wrapped with foil and shaken on an orbital shaker for 15 min after which the absorbance was read at 620 nm.

### Cell counting assay

LS8 or LS8-*NSD2* cells were seeded in quadruplicates for each harvesting time points in 60 mm dishes at 10^5^ cells/dish and cultured with DMEM (10% FBS and 1% penicillin/streptomycin). Cells were trypsinized and suspended with 1.5 to 2.5 ml culture medium, followed by cell counting using a hemocytometer at 24 h, 48 h, 72 h, 96 h, and 120 h post seeding.

### Real-time quantitative reverse transcription PCR

Total RNA was isolated from cells or mouse mandible tissues using miRNeasy Mini Kit (Qiagen) or standard RNA preparation protocol. Reverse transcription and quantitative real-time PCR for microRNA detection were carried out with miScript PCR system (Qiagen) according to the manufacturer’s protocol. Reverse transcription and quantitative real-time PCR for mRNAs were performed using a TaKaRa kit (TaKaRa, RR036A, RR420L). All Ct numbers were below 35 cycles. PCR products were examined by melting curve analysis and the sequences were confirmed. Fold change was calculated using the 2^–ΔΔCT^ method. The primers used for qPCR are listed in Table 2. The primers used to detect *miR-23a/b-3p* and *miR-24-1/2* were purchased from Qiagen.

**Table 2:** List of primers used for quantitative RT-PCR

Gene	Forward primer (5′-3′)	Reverse primer (5′-3′)
β-Actin	CTCTTCCAGCCTTCCTTC	ATCTCCTTCTGCATCCTGTC
Whsc1	TGCCAAAAAGGAGTACGTGTG	CTTCGGGAAAGTCCAAGGCAG
Pitx2	CTGGAAGCCACTTTCCAGAG	AAGCCATTCTTGCACAGCTC
PMIS-miR-23a	CTAAGGAAATCCCTGATCAGCAATGTGAT	GTCAGCTCTTAGTATTCATGAGATG
PMIS-miR-24	CTAACTGTTCCTGCTGATCAAACTGAGCCA	GTCAGCTCTTAGTATTCATGAGATG

### Western blot assays

Cell lysates from LS-8, HEK 293 cells and stable cell lines were analyzed on 4% stacking gel and 10 to 15% SDS-PAGE separating gels. Following electrophoresis, the protein was transferred to polyvinylidene fluoride membranes (Millipore), immunoblotted, and detected with a horseradish peroxidase-conjugated secondary antibody and Clarity Western ECL Blotting Substrate (Bio-Rad). The following antibodies were used to detect the proteins (Table 3).

**Table 3:** List of antibodies used for Western blot assays

Antibody Name	Manufacturer	Working Dilution
Anti-PITX2^ABC^ antibody PA1020	Capra Science	(WB) 1:2000
Anti-WHSC1/NSD2 mouse antibody [29D1] ab75359	Abcam	(WB) 1:2000
Anti-GAPDH mouse antibody sc32233	Santa Cruz Biotechnology	(WB) 1:10,000
Anti-Histone 3 rabbit antibody 9715	Cell Signaling	(WB) 1:2000
Anti-Histone H3 (mono methyl K36) rabbit antibodyab9048	Abcam	(WB) 1:2000

### Chromatin immunoprecipitation assay

ChIP assays were performed as previously described (33) using the ChIP Assay Kit (Zymo research). LS-8 cells were cross-linked in 1% formaldehyde at room temp for 7 min. Cross-linked cells were sonicated three times (6 s duration for each round, 25% of maximum amplitude) to shear the genomic DNA in to 200 to 1000 bp fragments. Then the DNA/protein complexes were immunoprecipitated with 5 μg PITX2 antibody (Capra Science), WHSC1 and H3K36me2 (Abcam) antibodies or 5 μg rabbit IgG as control. Precipitated DNAs were subjected to PCR to evaluate the enrichment of Pitx2 binding. The primers used for PCR are listed in Table 4. All the PCR products were analyzed on a 1.5% agarose gel for the correct size and confirmed by sequencing.

**Table 4:** List of primers used for ChIP-PCR assay

ChIP primers	Forward primer (5′-3′)	Reverse primer (5′-3′)
Whsc1 (Pitx2)-1	GAGCGATTCTCCTGCCTCAGCC	CACTTTGGGAGGCTGAGGCG
Whsc1 (con)-1	GGTGACTGTTGTTGTCCATAGC	GGTGGGAAGAGTTAAGCATCAC
Whsc1 (Pitx2)-2	ACATGTCTGCTGGTAACAAC	CTAAAACTCAAAGGGCTTGC
Whsc1 (con)-2	AACTCTGCACTTGGCAGGAA	TTGGCTTTGTGGGGCATGTA
Pitx2 (Pitx2)	TTCTGCCGATCCTTGTGGAC	TTCTGCCGATCCTTGTGGAC
Pitx2 (con)	TGGTCTTCAGCACCAAAGCG	TATTAGCCGGTAGCCCCAAC
pre-miR-23a-27a-24-2 (Pitx2)	TCCTGCCCTAACCTGTCAGA	AGCTAAGGACCCAACCGACT
pre-miR-23a-27a-24-2 (con)	GCCTCCCTGTTTGATGTCTC	CAGCTGGTTCTGTCATGCTC.
pre-miR-23b-27b-24-1 (Pitx2)	GAGCTGAGACCTGCTCATCC	GGTGACTGACTGTCCTGTGC
pre-miR-23b-27b-24-1 (con)	TGTGTGTGTGTGATGTTTAAGGA	CAGCTTTCTTTCTGTGTCAATGAT
Amelogenin (Pitx2)	GACTGCCTTTTAGTTCCATTCTC	TCTGTGATCCATATTTACACACCTG
Amelogenin (con)	CAGATCTTATTTGCAGCCTGA	AAAAGACATCTGCCCTCTTCT
Sox2 (Pitx2)	GAGCTTCTTTCCGTTGATGC	TTCCCTACTCCACCAACCTG
Sox2 (con)	TGGTCTTCAGCACCAAAGCG	TATTAGCCGGTAGCCCCAAC

### miRNA microarray

Incisor and molar tooth germs were dissected from P0 and P10 mice using a dissection microscope. To separate epithelium and mesenchyme, the tooth germs were treated with Dispase II and Collagenase I (Worthington) for 30 min at 37 °C. This procedure separates the epithelium from the mesenchyme and allows for specific RNA extraction of the two tissue types (37). Total RNAs including microRNA were prepared using miRNeasy Mini Kit from Qiagen. LC Sciences performed the miRNA microarray analyses.

### Statistical analysis

All quantified results are presented as mean ± SEM, and with an N value indicating the number of biological replicates. A two-tailed unpaired Student's *t* test and either one- or two-way ANOVA were used to determine statistical significance.

## Data availability

All data are available in the main article or the supporting information.

## Supporting information

This article contains supporting information.

## Conflict of interest

B. A. A. is the CEO of NaturemiRI. LLC.

## Supporting information

Supporting Figure S1 **Whsc1 expression during mouse molar development and activation of cell proliferation.***A* and *B*, Whsc1 immunofluorescence signal in mouse molars (dental epithelium outlined with *dashes*) at E11.5, E13.5, E14.5, E16.5 and P1. DAPI staining represents nuclei. Scale bar, 100 μm. *C*, MTT assays of LS-8 and LS-8-*NSD2* at 6 h, 24 h and 48 h post seeding at 20,000 cells in each well of 96-well plate (N = 3). *D*, cell counting assays of LS-8 and LS-8-*NSD2* at 24 h, 48 h, 72 h, 96 h and 120 h post seeding at 10^5^ cells in 60 mm dish (N = 4). ∗*p* < 0.05; ∗∗∗∗*p* < 0.0001. CL, cervical loop; DE, dental epithelium; DM, dental mesenchyme; Md, mandible; Mx, maxilla; SR, stellate reticulum.

Supporting Figure S2 **H3K36me2 is associated with the Pitx2 binding sites in *Whsc1* and *Pitx2* promoter.***A*, schematic of the *Whsc1* promoter chromatin region and ChIP primers. ChIP assay using anti-H3K36me2 Ab for chromatin immunoprecipitations. H3K36me2 was associated with the Pitx2 binding site in the *Whsc1* promoter. IgG alone did not IP the chromatin. PCR and no AB control groups did not produce a band. Control primers to an upstream region of the *Whsc1* promoter did not detect an IP product in any group except the input. qPCR products were analyzed for fold enrichment. *B*, schematic of the *Pitx2* promoter chromatin region and ChIP primers. ChIP assay using anti-H3K36me2 Ab for chromatin immunoprecipitations. H3K36me2 was associated with the Pitx2 binding site in the *Pitx2* promoter. IgG alone did not IP the chromatin. PCR and no AB control groups did not produce a band. Control primers to an upstream region of the *Pitx2* promoter did not detect an IP product in any group except the ChIP-H3K36me2 and input. qPCR products were analyzed for fold enrichment. All PCR bands were sequenced to confirm their specificity. ChIP, chromatin immunoprecipitation.

Supporting Figure S3 **Whsc1 is associated with the promoters of *Pitx2* downstream targets.***A*, schematic of the *Amelogenin* promoter chromatin region and ChIP primers. ChIP assay using either anti-PITX2^ABC^ or anti-WHSC1 Ab for chromatin immunoprecipitations. Whsc1 and Pitx2 bound to the *Amelogenin* promoter. IgG alone did not IP the chromatin. PCR and no AB control groups did not produce a band. Control primers to an upstream region of the *Amelogenin* promoter did not detect an IP product in any group except the input. *B*, ChIP assay using anti-H3K36me2 Ab for chromatin immunoprecipitations. H3K36me2 was associated with the Pitx2 binding site in the *Amelogenin* promoter. IgG alone did not IP the chromatin. PCR and no AB control groups did not produce a band. Control primers to an upstream region of the *Amelogenin* promoter did not detect an IP product in any group except the input. qPCR products were analyzed for fold enrichment. *C*, schematic of the *Sox2* promoter chromatin region and ChIP primers. Whsc1 and Pitx2 bound to the *Sox2* promoter. IgG alone did not IP the chromatin. PCR and no AB control groups did not produce a band. Control primers to an upstream region of the *Sox2* promoter did not detect an IP product in any group except the input. *D*, ChIP assay using anti-H3K36me2 Ab for chromatin immunoprecipitations. H3K36me2 was associated with the Pitx2 binding site in the *Sox2* promoter. IgG alone did not IP the chromatin. PCR and no AB control groups did not produce a band. Control primers to an upstream region of the *Sox2* promoter did not detect an IP product in any group except the input. qPCR products were analyzed for fold enrichment. All PCR bands were sequenced to confirm their specificity. ChIP, chromatin immunoprecipitation.

Supporting Figure S4 **Pitx2 binds to the upstream distal region of *pre-miR-23b-27b-24-1*.***A*, schematic representation and location of the Pitx2 binding site in the *pre-miR-23b-27b-24-1* promoter. Pound sign indicates the region containing a conserved Pitx2 binding element (TCATCC). The *gray line* (con) indicates a 5′ region which lacks Pitx2 consensus binding motif and was used as negative control. *B*, the Pitx2 binding element of the mouse pre-*miR-23b-27b-24-1* promoter was mapped to a highly conserved region among monkey, dog, human, and rat. The *red box* indicates the PCR amplified region on pre-*miR-23b-27b-24-1* promoter in *A*). *C*, ChIP-PCR assay of endogenous Pitx2 binding to the chromatin region approximately 73 kb upstream of pre-*miR-23b-27b-24-1* transcript in LS-8 cells (see *asterisk*). *D*, control ChIP-PCR assay using the Pitx2 antibody and primers to an upstream region of the pre-*miR-23b-27b-24-1* transcript. This chromatin region does not contain a Pitx2 binding site. ChIP, chromatin immunoprecipitation.

Supporting Figure S5 ***PMIS-miR-23* and *PMIS-miR-24* specifically inhibit their miRs.***A*, RNA isolated from the LS-8-*PMIS-EV* and LS-8-*PMIS-miR-23* cells were analyzed by qPCR for *miR-23a/b*, *miR-24-1/2* and *miR-27a/b* levels. Relative fold changes normalized to U6 of each miR are shown (N = 3). *B*, RNA isolated from the LS-8-*PMIS-EV* and LS-8-*PMIS-miR-24* cells were analyzed by qPCR for *miR-23a/b*, *miR-24-1/2* and *miR-27a/b* levels. Relative fold changes normalized to U6 of each miR are shown (N = 4).

Supporting Figure S6 ***miR-23* and *miR-24* negatively regulate H3K36 methylation.***A*, Western blot of H3K36me1 in LS-8-*PMIS-EV*, LS-8-*PMIS-miR-23* and LS-8-*PMIS-miR-24* stable cell lines. Histone 3 (H3) is shown as a loading control. *B*, Western blot of H3K36me2 in LS-8 cells transfected with *PMIS-EV*, *PMIS-miR-23* and *PMIS-miR-24* constructs. Histone 3 (H3) is shown as a loading control.
