# Supplementary material for: Wolf-Hirschhorn syndrome candidate 1 (Whsc1) methyltransferase signals via a Pitx2-miR-23/24 axis to effect tooth development
Source: J Biol Chem. 2023 Oct 6;299(11):105324. doi: 10.1016/j.jbc.2023.105324 (PMC10656234; doi:10.1016/j.jbc.2023.105324)
Supplement: Supporting Figure S1 — Whsc1 expression during mouse molar development and activation of cell proliferation.A and B, Whsc1 immunofluorescence signal in mouse molars (dental epithelium outlined with dashes) at E11.5, E13.5, E14.5, E16.5 and P1. DAPI staining represents nuclei. Scale bar, 100 μm. C, MTT assays of LS-8 and LS-8-NSD2 at 6 h, 24 h and 48 h post seeding at 20,000 cells in each well of 96-well plate (N = 3). D, cell counting assays of LS-8 and LS-8-NSD2 at 24 h, 48 h, 72 h, 96 h and 120 h post seeding at 105 cells in 60 mm dish (N = 4). ∗p < 0.05; ∗∗∗∗p < 0.0001. CL, cervical loop; DE, dental epithelium; DM, dental mesenchyme; Md, mandible; Mx, maxilla; SR, stellate reticulum. [file mmc1.pptx]

## Slide 1
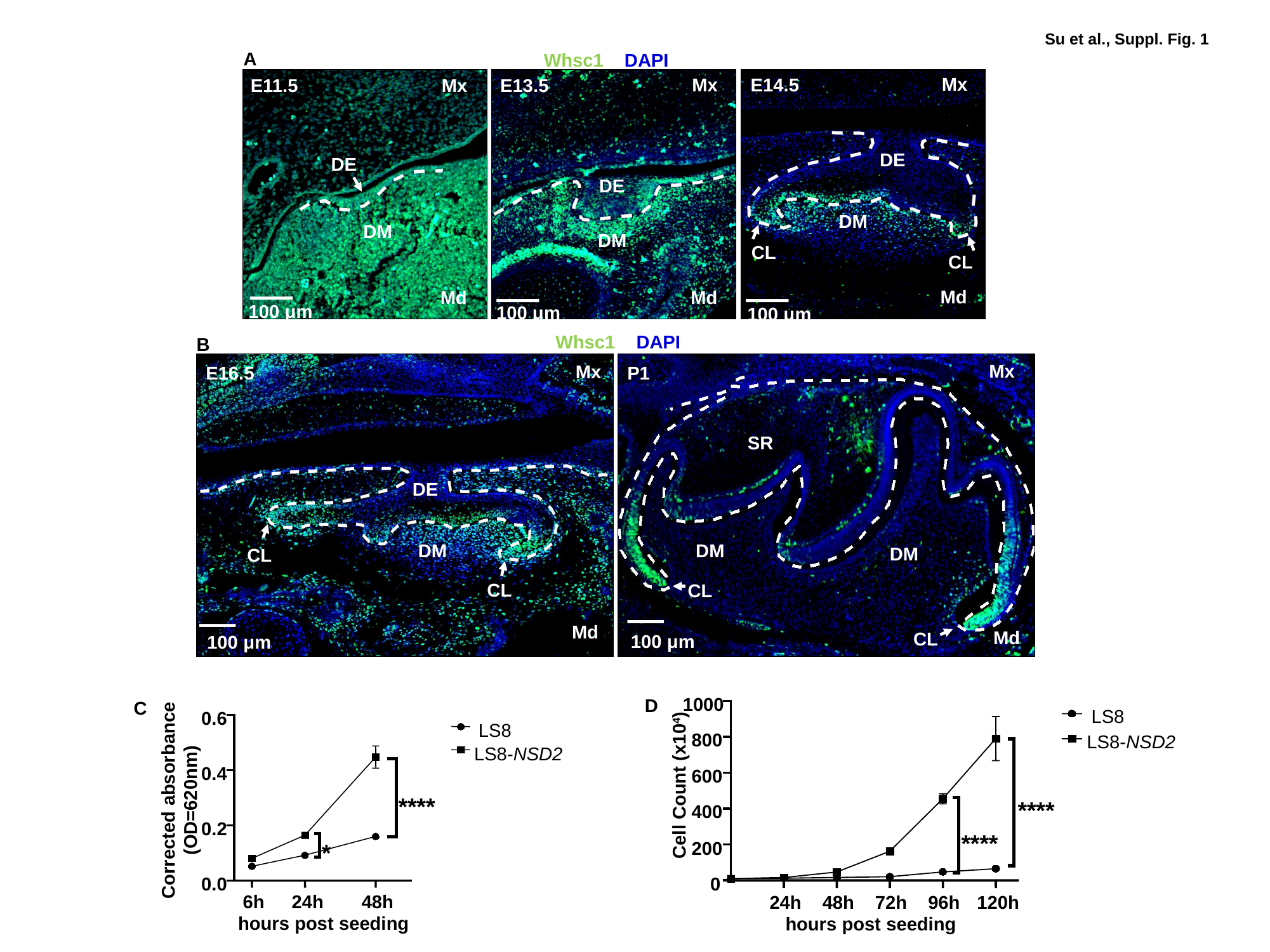

Su et al., Suppl. Fig. 1
A
Whsc1 DAPI
Mx
Mx
E14.5
E11.5
E13.5
Mx
DE
DE
DE
DM
DM
DM
CL
CL
Md
Md
Md
100 μm
100 μm
100 μm
Whsc1 DAPI
B
Mx
Mx
E16.5
P1
SR
DE
DM
DM
DM
CL
CL
CL
Md
Md
CL
100 μm
100 μm
D
C
1000
LS8
800
LS8-NSD2
600
Cell Count (x104)
****
400
****
200
0
24h
48h
72h
96h
120h
hours post seeding
0.6
LS8
LS8-NSD2
0.4
Corrected absorbance
(OD=620nm)
****
0.2
*
0.0
6h
24h
48h
hours post seeding
