# Supplementary material for: Wolf-Hirschhorn syndrome candidate 1 (Whsc1) methyltransferase signals via a Pitx2-miR-23/24 axis to effect tooth development
Source: J Biol Chem. 2023 Oct 6;299(11):105324. doi: 10.1016/j.jbc.2023.105324 (PMC10656234; doi:10.1016/j.jbc.2023.105324)
Supplement: Supporting Figure S2 — H3K36me2 is associated with the Pitx2 binding sites in Whsc1 and Pitx2 promoter.A, schematic of the Whsc1 promoter chromatin region and ChIP primers. ChIP assay using anti-H3K36me2 Ab for chromatin immunoprecipitations. H3K36me2 was associated with the Pitx2 binding site in the Whsc1 promoter. IgG alone did not IP the chromatin. PCR and no AB control groups did not produce a band. Control primers to an upstream region of the Whsc1 promoter did not detect an IP product in any group except the input. qPCR products were analyzed for fold enrichment. B, schematic of the Pitx2 promoter chromatin region and ChIP primers. ChIP assay using anti-H3K36me2 Ab for chromatin immunoprecipitations. H3K36me2 was associated with the Pitx2 binding site in the Pitx2 promoter. IgG alone did not IP the chromatin. PCR and no AB control groups did not produce a band. Control primers to an upstream region of the Pitx2 promoter did not detect an IP product in any group except the ChIP-H3K36me2 and input. qPCR products were analyzed for fold enrichment. All PCR bands were sequenced to confirm their specificity. ChIP, chromatin immunoprecipitation. [file mmc2.pptx]

## Slide 1
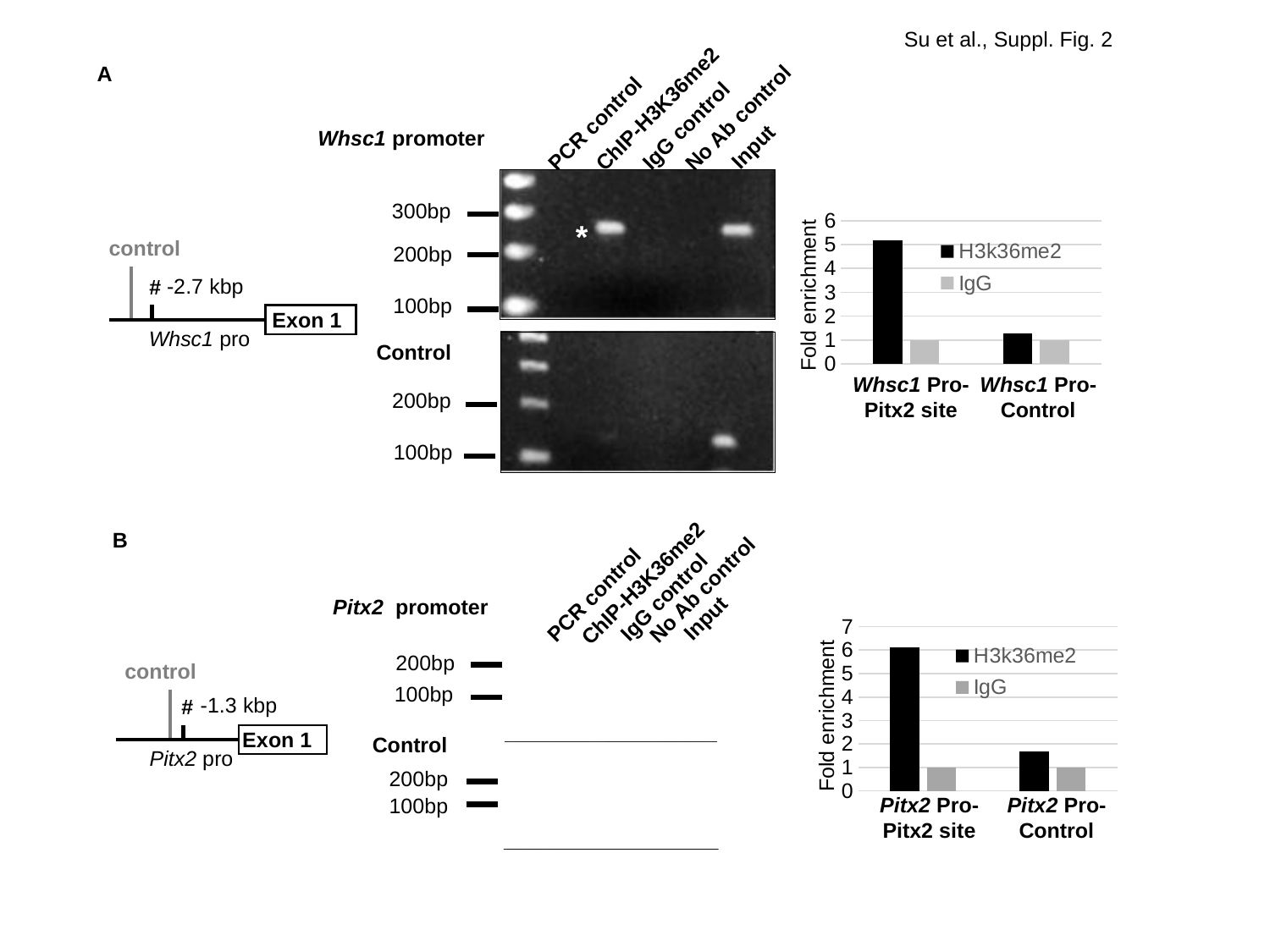

Su et al., Suppl. Fig. 2
A
ChIP-H3K36me2
No Ab control
IgG control
PCR control
Whsc1 promoter
Input
300bp
### Chart
| Category | H3k36me2 | IgG |
|---|---|---|
| Whsc1pr Ptx2 | 5.187358218604039 | 1.0 |
| Whc1pr Cntr | 1.2834258975629036 | 1.0 |Fold enrichment
Whsc1 Pro-
Pitx2 site
Whsc1 Pro-
Control
*
control
-2.7 kbp
#
Exon 1
 Whsc1 pro
200bp
100bp
Control
200bp
100bp
B
ChIP-H3K36me2
No Ab control
PCR control
IgG control
Pitx2 promoter
Input
### Chart
| Category | H3k36me2 | IgG |
|---|---|---|
| Pitx2pr Pitx2 | 6.12610972694737 | 1.0 |
| Pitx2pr cntr | 1.69133979692753 | 1.0 |Fold enrichment
Pitx2 Pro-
Pitx2 site
Pitx2 Pro-
Control
200bp
control
-1.3 kbp
#
Exon 1
 Pitx2 pro
*
100bp
Control
200bp
100bp
