# Supplementary material for: Wolf-Hirschhorn syndrome candidate 1 (Whsc1) methyltransferase signals via a Pitx2-miR-23/24 axis to effect tooth development
Source: J Biol Chem. 2023 Oct 6;299(11):105324. doi: 10.1016/j.jbc.2023.105324 (PMC10656234; doi:10.1016/j.jbc.2023.105324)
Supplement: Supporting Figure S3 — Whsc1 is associated with the promoters of Pitx2 downstream targets.A, schematic of the Amelogenin promoter chromatin region and ChIP primers. ChIP assay using either anti-PITX2ABC or anti-WHSC1 Ab for chromatin immunoprecipitations. Whsc1 and Pitx2 bound to the Amelogenin promoter. IgG alone did not IP the chromatin. PCR and no AB control groups did not produce a band. Control primers to an upstream region of the Amelogenin promoter did not detect an IP product in any group except the input. B, ChIP assay using anti-H3K36me2 Ab for chromatin immunoprecipitations. H3K36me2 was associated with the Pitx2 binding site in the Amelogenin promoter. IgG alone did not IP the chromatin. PCR and no AB control groups did not produce a band. Control primers to an upstream region of the Amelogenin promoter did not detect an IP product in any group except the input. qPCR products were analyzed for fold enrichment. C, schematic of the Sox2 promoter chromatin region and ChIP primers. Whsc1 and Pitx2 bound to the Sox2 promoter. IgG alone did not IP the chromatin. PCR and no AB control groups did not produce a band. Control primers to an upstream region of the Sox2 promoter did not detect an IP product in any group except the input. D, ChIP assay using anti-H3K36me2 Ab for chromatin immunoprecipitations. H3K36me2 was associated with the Pitx2 binding site in the Sox2 promoter. IgG alone did not IP the chromatin. PCR and no AB control groups did not produce a band. Control primers to an upstream region of the Sox2 promoter did not detect an IP product in any group except the input. qPCR products were analyzed for fold enrichment. All PCR bands were sequenced to confirm their specificity. ChIP, chromatin immunoprecipitation. [file mmc3.pptx]

## Slide 1
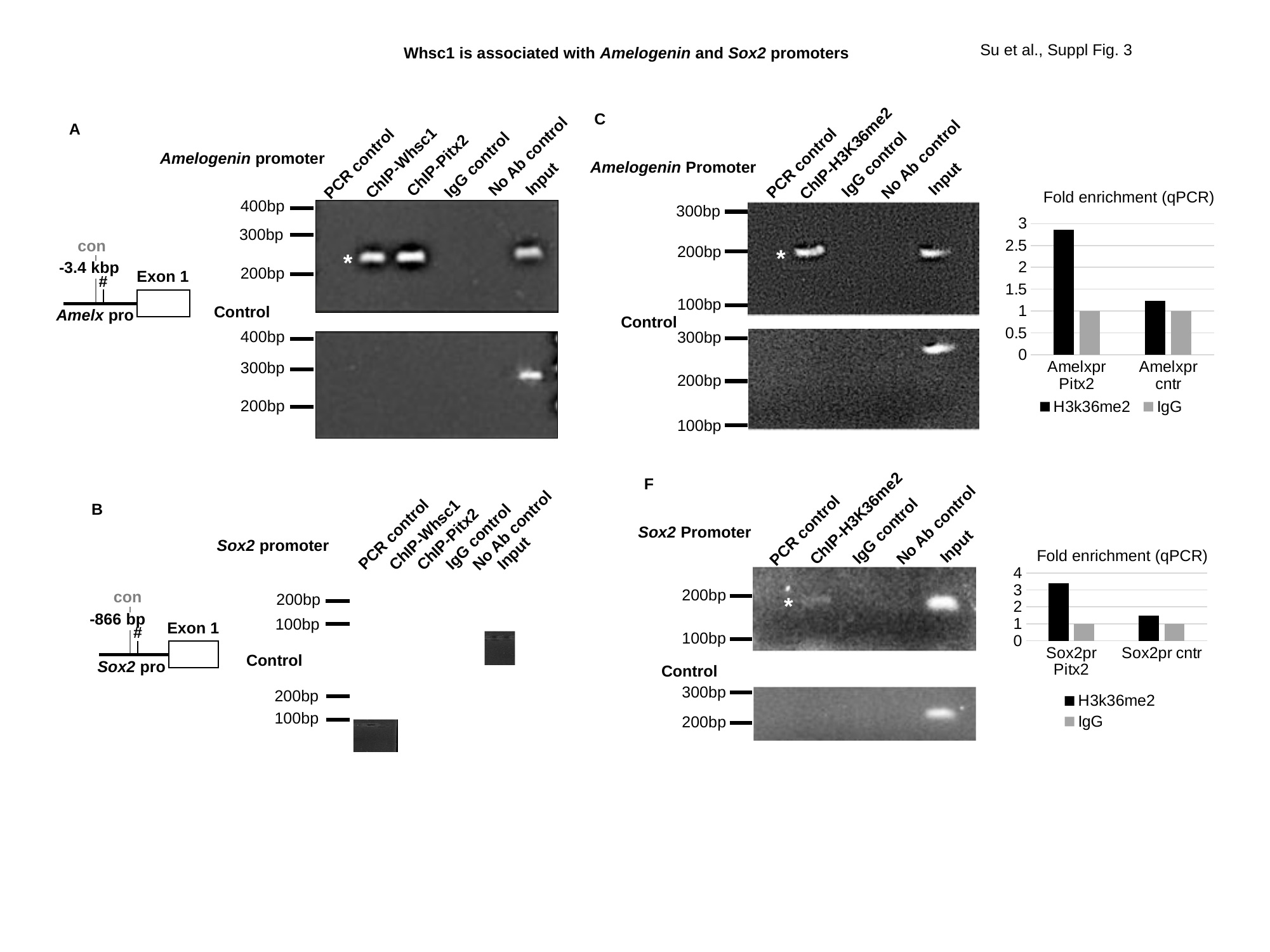

Su et al., Suppl Fig. 3
Whsc1 is associated with Amelogenin and Sox2 promoters
C
ChIP-H3K36me2
No Ab control
PCR control
IgG control
Amelogenin Promoter
Input
300bp
200bp
100bp
Control
300bp
200bp
100bp
A
No Ab control
Amelogenin promoter
ChIP-Whsc1
PCR control
IgG control
ChIP-Pitx2
Input
400bp
300bp
200bp
Control
400bp
300bp
200bp
con
-3.4 kbp
Exon 1
#
 Amelx pro
Fold enrichment (qPCR)
### Chart
| Category | H3k36me2 | IgG |
|---|---|---|
| Amelxpr Pitx2 | 2.85317612378074 | 1.0 |
| Amelxpr cntr | 1.23701758388567 | 1.0 |*
*
F
B
No Ab control
PCR control
ChIP-Whsc1
ChIP-Pitx2
IgG control
Sox2 promoter
Input
con
-866 bp
Exon 1
#
 Sox2 pro
200bp
*
100bp
Control
200bp
100bp
ChIP-H3K36me2
No Ab control
IgG control
PCR control
Sox2 Promoter
Input
Fold enrichment (qPCR)
### Chart
| Category | H3k36me2 | IgG |
|---|---|---|
| Sox2pr Pitx2 | 3.386981249450104 | 1.0 |
| Sox2pr cntr | 1.4742692172910963 | 1.0 |
200bp
*
100bp
Control
300bp
200bp
