# Supplementary material for: Wolf-Hirschhorn syndrome candidate 1 (Whsc1) methyltransferase signals via a Pitx2-miR-23/24 axis to effect tooth development
Source: J Biol Chem. 2023 Oct 6;299(11):105324. doi: 10.1016/j.jbc.2023.105324 (PMC10656234; doi:10.1016/j.jbc.2023.105324)
Supplement: Supporting Figure S4 — Pitx2 binds to the upstream distal region of pre-miR-23b-27b-24-1.A, schematic representation and location of the Pitx2 binding site in the pre-miR-23b-27b-24-1 promoter. Pound sign indicates the region containing a conserved Pitx2 binding element (TCATCC). The gray line (con) indicates a 5′ region which lacks Pitx2 consensus binding motif and was used as negative control. B, the Pitx2 binding element of the mouse pre-miR-23b-27b-24-1 promoter was mapped to a highly conserved region among monkey, dog, human, and rat. The red box indicates the PCR amplified region on pre-miR-23b-27b-24-1 promoter in A). C, ChIP-PCR assay of endogenous Pitx2 binding to the chromatin region approximately 73 kb upstream of pre-miR-23b-27b-24-1 transcript in LS-8 cells (see asterisk). D, control ChIP-PCR assay using the Pitx2 antibody and primers to an upstream region of the pre-miR-23b-27b-24-1 transcript. This chromatin region does not contain a Pitx2 binding site. ChIP, chromatin immunoprecipitation. [file mmc4.pptx]

## Slide 1
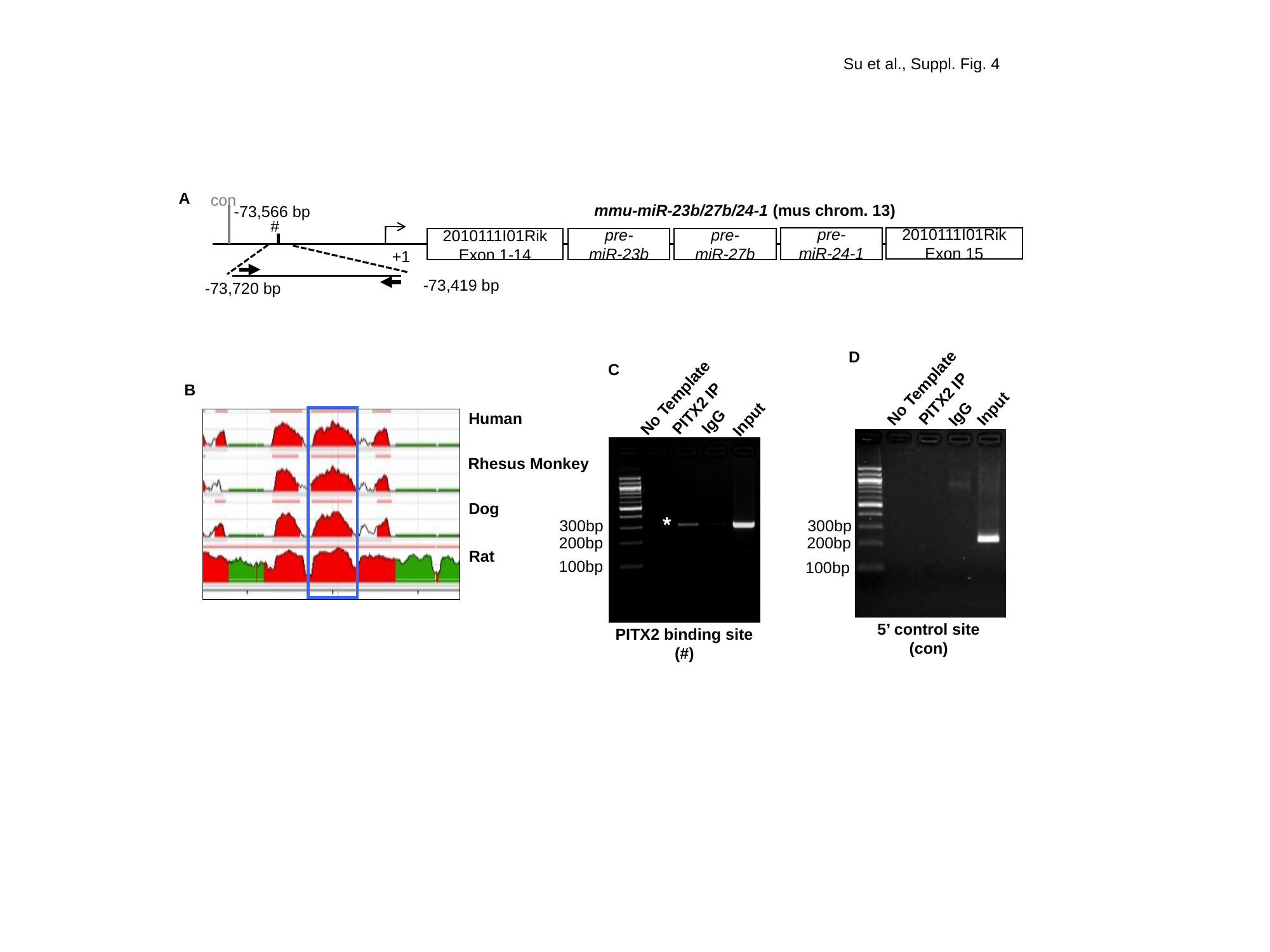

Su et al., Suppl. Fig. 4
A
con
mmu-miR-23b/27b/24-1 (mus chrom. 13)
-73,566 bp
#
2010111I01Rik
Exon 15
pre-
miR-24-1
pre-
miR-27b
pre-
miR-23b
2010111I01Rik
Exon 1-14
+1
-73,419 bp
-73,720 bp
D
No Template
PITX2 IP
Input
IgG
5’ control site
(con)
300bp
200bp
100bp
No Template
PITX2 IP
Input
IgG
*
PITX2 binding site
(#)
C
300bp
200bp
100bp
B
Human
Rhesus Monkey
Dog
Rat
