# Supplementary material for: Wolf-Hirschhorn syndrome candidate 1 (Whsc1) methyltransferase signals via a Pitx2-miR-23/24 axis to effect tooth development
Source: J Biol Chem. 2023 Oct 6;299(11):105324. doi: 10.1016/j.jbc.2023.105324 (PMC10656234; doi:10.1016/j.jbc.2023.105324)
Supplement: Supporting Figure S5 — PMIS-miR-23 and PMIS-miR-24 specifically inhibit their miRs.A, RNA isolated from the LS-8-PMIS-EV and LS-8-PMIS-miR-23 cells were analyzed by qPCR for miR-23a/b, miR-24-1/2 and miR-27a/b levels. Relative fold changes normalized to U6 of each miR are shown (N = 3). B, RNA isolated from the LS-8-PMIS-EV and LS-8-PMIS-miR-24 cells were analyzed by qPCR for miR-23a/b, miR-24-1/2 and miR-27a/b levels. Relative fold changes normalized to U6 of each miR are shown (N = 4). [file mmc5.pptx]

## Slide 1
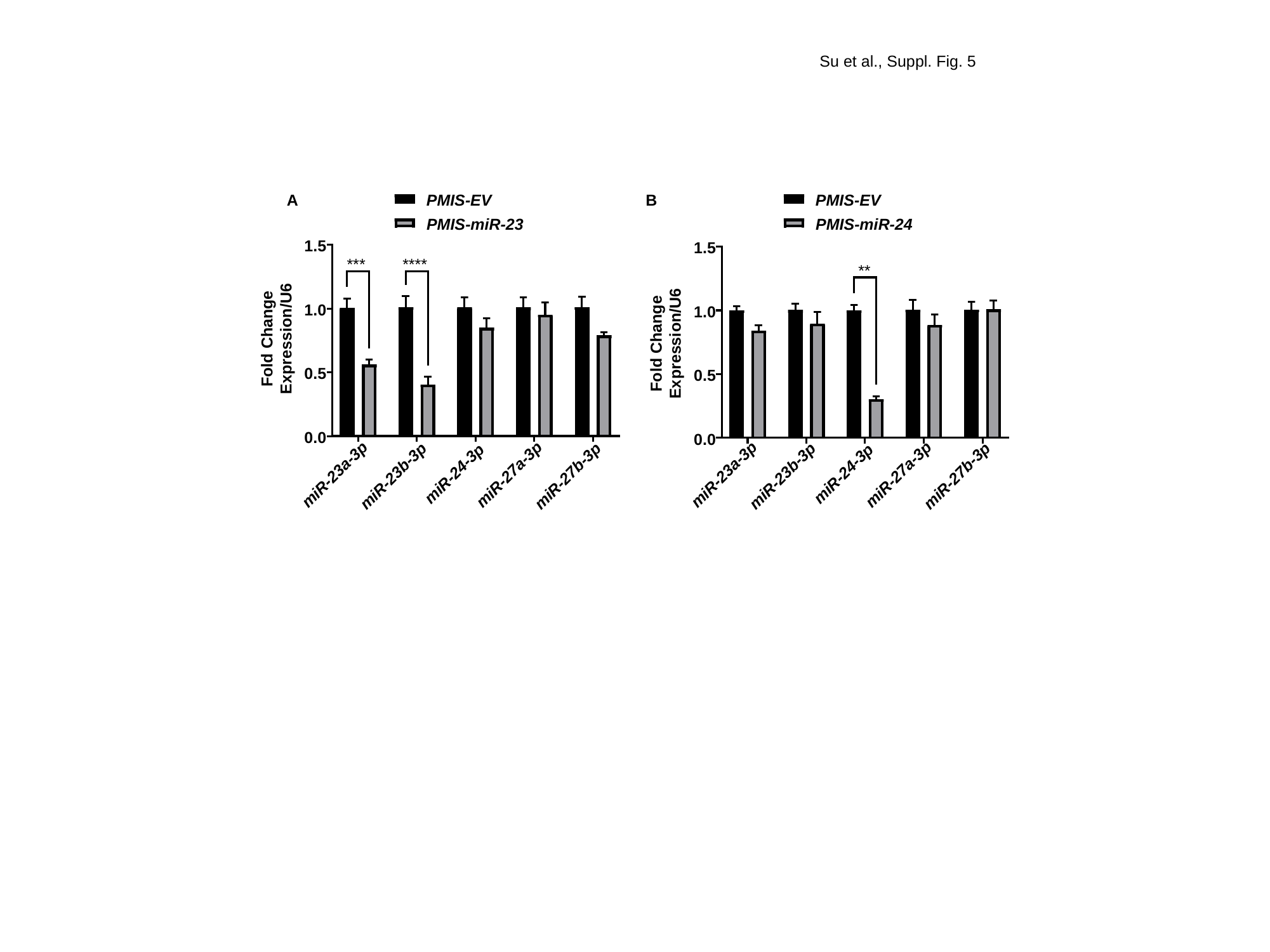

Su et al., Suppl. Fig. 5
A
B
PMIS-EV
PMIS-miR-24
1.5
**
1.0
Fold Change Expression/U6
0.5
0.0
miR-24-3p
miR-23a-3p
miR-27a-3p
miR-23b-3p
miR-27b-3p
PMIS-EV
PMIS-miR-23
1.5
***
****
1.0
0.5
0.0
Fold Change Expression/U6
miR-24-3p
miR-23a-3p
miR-27a-3p
miR-23b-3p
miR-27b-3p
