# Supplementary material for: Wolf-Hirschhorn syndrome candidate 1 (Whsc1) methyltransferase signals via a Pitx2-miR-23/24 axis to effect tooth development
Source: J Biol Chem. 2023 Oct 6;299(11):105324. doi: 10.1016/j.jbc.2023.105324 (PMC10656234; doi:10.1016/j.jbc.2023.105324)
Supplement: Supporting Figure S6 — miR-23 and miR-24 negatively regulate H3K36 methylation.A, Western blot of H3K36me1 in LS-8-PMIS-EV, LS-8-PMIS-miR-23 and LS-8-PMIS-miR-24 stable cell lines. Histone 3 (H3) is shown as a loading control. B, Western blot of H3K36me2 in LS-8 cells transfected with PMIS-EV, PMIS-miR-23 and PMIS-miR-24 constructs. Histone 3 (H3) is shown as a loading control. [file mmc6.pptx]

## Slide 1
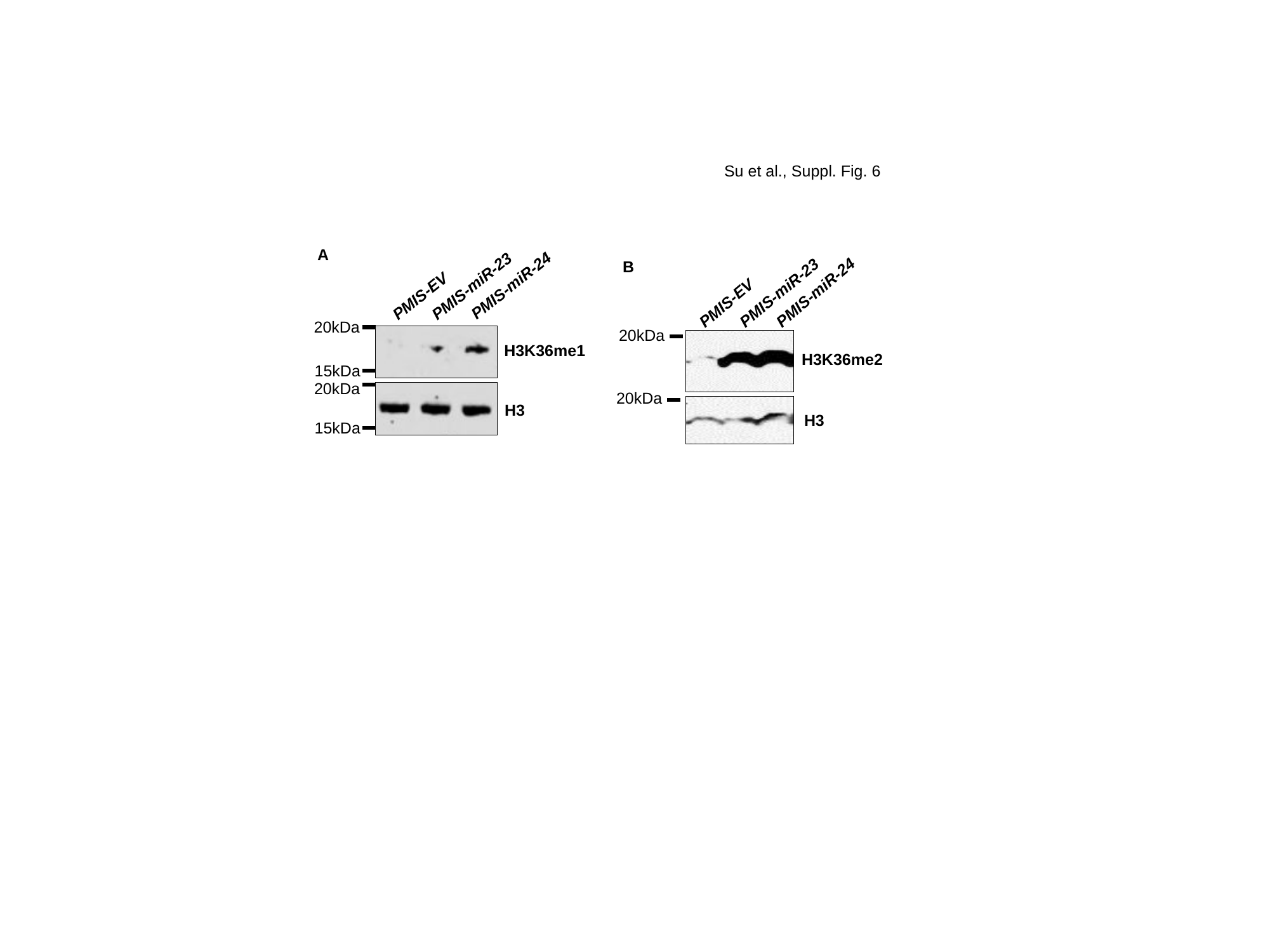

Su et al., Suppl. Fig. 6
A
B
PMIS-miR-24
PMIS-miR-23
PMIS-EV
20kDa
H3K36me1
15kDa
20kDa
15kDa
H3
PMIS-miR-23
PMIS-miR-24
PMIS-EV
20kDa
H3K36me2
20kDa
H3
